# Associated minerals in chrysotile deposits and their potential health risks

**DOI:** 10.3389/fpubh.2025.1583469

**Published:** 2025-05-07

**Authors:** Eric J. Chatfield

**Affiliations:** Chatfield Technical Consulting Limited, Mississauga, ON, Canada

**Keywords:** chrysotile, UICC, tremolite, actinolite, balangeroite, diopside, fiber, asbestos

## Abstract

Chrysotile samples from different deposits and the UICC-A and UICC-B standards have been analyzed by a procedure in which the chrysotile is removed by successive treatments in hydrochloric acid and sodium hydroxide, followed by transmission electron microscopy (TEM) examination of the residues. Two separate TEM fiber counts of a minimum of 100 fibers each were made for each sample: fibers longer than 5 μm and fibers with lengths between 0.5 μm and 5 μm. The tremolite/actinolite in each sample was quantified in terms of fibers/gram of chrysotile and as mass fraction in parts per million (ppm). Chrysotile from almost all of the commercial deposits examined was found to contain tremolite/actinolite. In particular, UICC-B Canadian chrysotile was found to contain an average of approximately 180 ppm tremolite/actinolite, equivalent to 1.25 × 10^7^ fibers per gram (longer than 5 μm) of tremolite/actinolite, a proportion of which is asbestiform. The results also showed that both the UICC-B and UICC-A chrysotile standards are contaminated by Amosite. The primary grades of chrysotile from Coalinga, United States, and Minaçu, Brazil, were found to contain substantially less than 1 ppm of tremolite/actinolite. An asbestiform variety of the pyroxene diopside was detected in chrysotile from the Balangero mine in Italy. The asbestiform diopside has a fiber concentration and mass fraction comparable to those of tremolite/actinolite in chrysotile from other sources. Some of the diopside fibers are considerably longer than the tremolite/actinolite fibers found in other sources of chrysotile. Low levels (<4 ppm) of tremolite/actinolite were detected in Balangero chrysotile. No fibers with compositions consistent with Balangeroite were detected. It was found that Balangeroite does not survive the acid-alkali dissolution procedure, and it probably has durability comparable to that of chrysotile. Publications that claim the absence of tremolite/actinolite in UICC-B chrysotile were based on analytical methods that had insufficient sensitivity. Use of these analytical methods permitted only a 1 in 5 chance that a single tremolite/actinolite fiber would be detected. The concentrations of tremolite/actinolite and Amosite found in the reference UICC chrysotile standards raise questions as to the validity of historical biological experiments carried out using these materials.

## 1 Introduction

When lung tissues of former miners and millers in the Quebec chrysotile industry were analyzed to determine the presence and concentrations of asbestos fibers, it was found that tremolite/actinolite was a major contributor to the mineral fiber content (1, 2). It was suggested that chrysotile was preferentially removed from the lungs and that other mineral fibers were retained (2). A study in 1995 and 1996 of mine and mill workers in the Thetford Mines area of Québec found that 88% of the mineral fibers found in the lungs of these workers were tremolite, and only 4% were identified as chrysotile (3, 4). It was hypothesized that chrysotile was not durable in lung tissue fluids and that chemical processes removed it, whereas tremolite/actinolite was resistant to attack by lung fluids and that it accumulated in the lungs throughout the period of airborne exposure. Furthermore, it was suggested that the tremolite/actinolite fibers could be responsible for mesothelioma and lung cancer found in these workers, rather than as a consequence of the much larger exposures to chrysotile (5–7). This suggestion became known as “the amphibole hypothesis” (8, 9), and was controversial, particularly in the legal community (10).

Much of the discussion concerning the presence of tremolite/actinolite in chrysotile has been based on the observation of these fibers in lung tissue (1–7). Only a few studies have been published in which amphibole fiber concentrations and fiber sizes have been determined in chrysotile from known sources (11, 12). A survey of the Jeffrey mine in Asbestos, Québec, reported in 2001, discussed the mechanisms and reactions by which the amphibole present in the deposit was formed, most of which was tremolite/actinolite (13). It was also reported that only a small fraction of the tremolite/actinolite was asbestiform. Millette et al. reported a measurement of approximately 94 ppm tremolite in chrysotile from Black Lake, Québec, and found no tremolite in two measurements made on chrysotile from Coalinga, CA (11). The analytical sensitivities of the two measurements on Coalinga chrysotile were 0.0002 ppm and 0.00007 ppm. However, in both analyses of Coalinga chrysotile it was noted that antigorite fibers were present.

UICC-A and UICC-B chrysotile are two of the reference materials prepared in the 1960’s for research on asbestos (14). The intent was to provide a large amount of well-characterized asbestos materials so that individual researchers were all using the same materials. UICC-B chrysotile is the only one to have been prepared from a mixture of sources. Each of the other UICC reference materials, including UICC-A chrysotile, was derived from a single source. UICC-A chrysotile was prepared using chrysotile from a mine in Zimbabwe (formerly Rhodesia).

UICC-B chrysotile was intended to be representative of Québec production at the time, so the weight incorporated from each mine was approximately proportional to the production from that mine. Although the identity of the contributing mines was not disclosed, it can be deduced that one mine that contributed 50% to the mixture was the Jeffrey Mine at Asbestos, Québec, because it was by far the largest chrysotile mine in the Québec mining region. For production of the first 1,120 lb. batch, 560 lbs. was from the Jeffrey Mine, and this was mixed with 80 lbs. from each of 7 other Québec mines.

Some of the publications on tremolite in chrysotile are contradictory. One study published in 1997 and 1998 (15, 16) claimed that UICC-B chrysotile is “uncontaminated by tremolite.” Claims that UICC-B chrysotile is “free of tremolite by electron microscopy” continue to be made as late as 2021 (17). Another publication in 2010 refers to chrysotile from the Carey mine in Quebec as “tremolite-free” (18). However, neither of these studies pays any attention to the limit of detection applicable to the analytical methods used. Concurrently, measurable tremolite/actinolite concentrations in commercial products manufactured from chrysotile have been reported for the purposes of product liability litigation (19–21). Since the majority of the chrysotile used in manufacture of these North American commercial products was from Québec, it is difficult to reconcile these measurements with the reported absence of tremolite in UICC-B chrysotile other than by use of a limit of detection argument.

In the present study, chrysotile samples from a number of different mine sources, UICC-A chrysotile and UICC-B chrysotile were analyzed by a procedure in which the chrysotile was dissolved by successive treatments in hydrochloric acid (HCl) and sodium hydroxide (NaOH). The residue after the dissolution treatment was examined by transmission electron microscopy (TEM). Where feasible, the lengths and widths of a minimum of 100 fibers longer than 5 μm were recorded, and also for a minimum of 100 fibers 0.5 μm–5 μm. This method is a modification of the procedure of Addison and Davies (22), in that hydrochloric acid is used instead of sulfuric acid, and TEM is used to quantify amphibole fibers in the residue, rather than x-ray diffraction.

## 2 Materials and methods

### 2.1 Materials

The samples analyzed were production grade commercial chrysotile from Canadian, United States, Brazil and Italian mines. Samples of UICC-A and UICC-B chrysotile from two separate sources were also analyzed. In some cases, a number of different grades of chrysotile were available, and these were analyzed separately.

There are important considerations when determining the weight of chrysotile to be used for each analysis:

(a) given that the residue remaining after the acid-alkali dissolution can be as low as 0.2%, it is important that the residue can be weighed accurately;
(b) the weight of chrysotile that can be considered representative with respect to its content of amphibole;
(c) the volumes and molarities of reagents to be used to ensure excess for the weight of chrysotile to be dissolved.

When available, approximately 1.5–1.9 g of chrysotile was used for each analysis. This ensured that for the samples that produced the lowest residue weights, a residue of several milligrams of residue was available for weighing. For most of the samples, somewhat larger residue weights were obtained. No information is available concerning the weight of chrysotile that can be considered representative in terms of amphibole content. That information can only be obtained by repeat measurements, but some of the analyses in this study appear to confirm that approximately 1.5 g is representative, at least for the chrysotile deposits that were analyzed. Approximately 80 mL of 2 M HCl and 80 mL of 4 M NaOH solution were calculated to represent an excess of reagents for the dissolution procedure.

### 2.2 Methods

#### 2.2.1 Chrysotile dissolution

All water used in the dissolution procedure was freshly distilled and pressure filtered through a 0.1 μm porosity mixed esters of cellulose (MEC) filter.

The chrysotile dissolution procedure consists of treatment in HCl according to the reaction:


$${\mathrm{Mg}}_{3}{Si}_{2}O_{5}{\left(OH\right)}_{4}+\mathrm{6HCl}\to {\mathrm{3MgCl}}_{2}+2H_{2}{SiO}_{3}$$

The silica gel that remains from the acid treatment is then dissolved in NaOH according to the reaction:


$$H_{2}{SiO}_{3}+\mathrm{2NaOH}\to {Na}_{2}{SiO}_{3}+2H_{2}O$$

**Figure 1:**
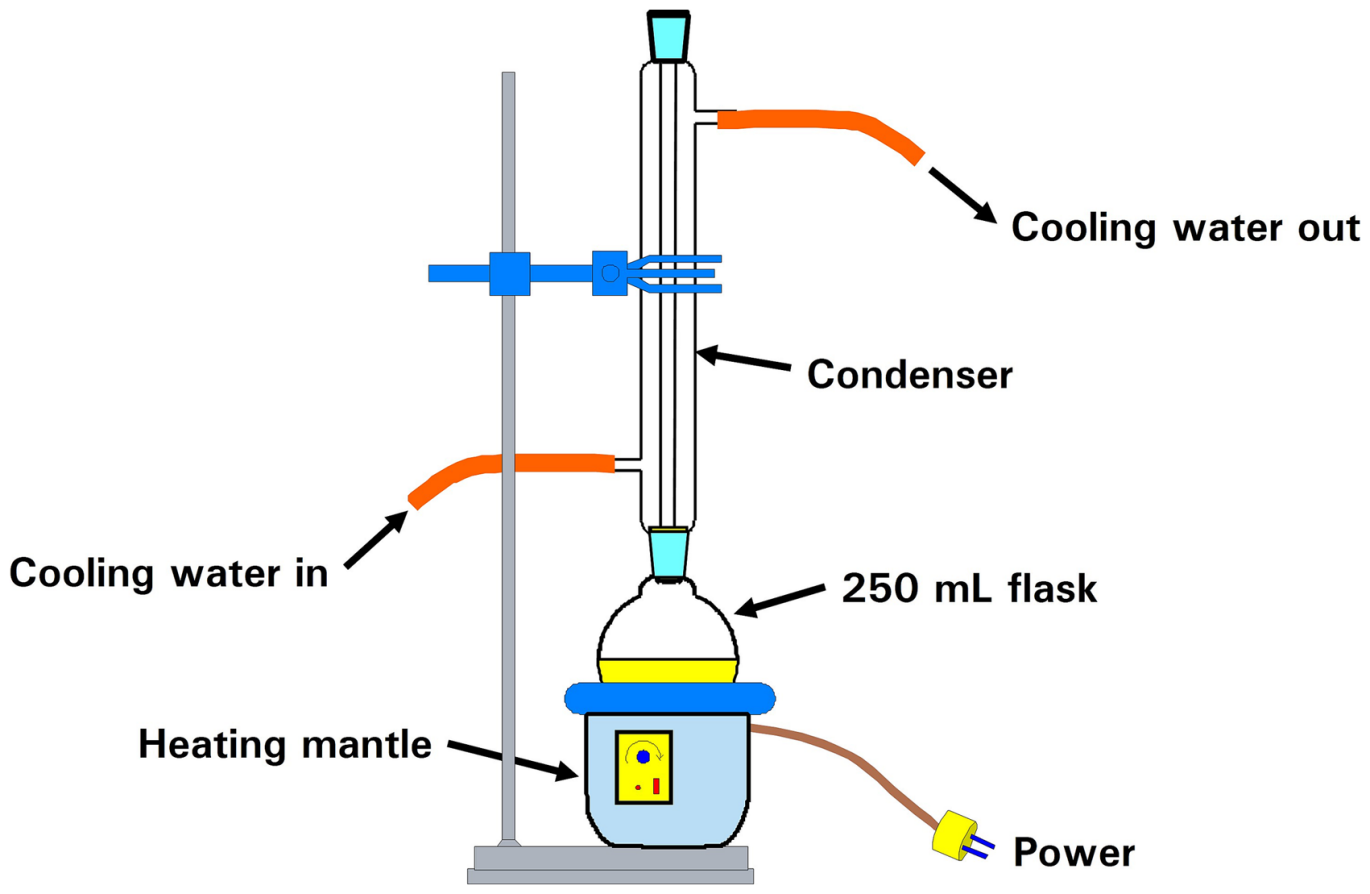
Reflux apparatus for acid/alkali dissolution of chrysotile.

The apparatus for the dissolution procedure is shown in Figure 1. Approximately 1.5 g of chrysotile is placed in the flask, and 80 mL of 2 M HCl is added. The flask is heated to boiling and allowed to reflux for approximately 1 h. The flask is allowed to cool, and the contents are transferred to a beaker. As much as possible of the acid and solids are transferred to four 15 mL centrifuge tubes. The tubes are centrifuged at 3600 rpm for 5 min. It is important that all traces of magnesium chloride be removed from the centrifugate before the treatment with NaOH. Using a 10 mL plastic pipette and rubber bulb, the supernatant acid is removed from the centrifuge tubes and discarded. The balance of the acid and solids are added to the centrifuge tubes, dispersed in filtered distilled water and the tubes are centrifuged again for 5 min. The supernatant acid is again removed from the centrifuge tubes and discarded. The centrifugate in each centrifuge tube is dispersed in filtered distilled water, and the tubes are centrifuged for 5 min. The supernatant liquid is removed and discarded. This procedure is repeated two more times. The centrifugate from all four centrifuge tubes is combined and returned to the flask. Approximately 80 mL of 4 M NaOH is added to the flask. The flask is heated to boiling and allowed to reflux for approximately 1 h.

After cooling, the contents of the flask are transferred to a beaker. As much as possible of the NaOH solution and solids are transferred to four 15 mL centrifuge tubes. The tubes are centrifuged at 3600 rpm for 5 min. Using a 10 mL plastic pipette and rubber bulb, the supernatant NaOH solution is removed from the centrifuge tubes and discarded. The balance of the NaOH solution and solids are added to the centrifuge tubes and the tubes are centrifuged again for 5 min. The supernatant NaOH solution is again removed from the centrifuge tubes and discarded. A 47 mm diameter glass filtration system is set up with a pre-weighed 0.2 μm pore size track-etched polycarbonate (PC) filter. Using filtered distilled water from a wash bottle, the centrifugate in each of the centrifuge tubes is combined in a beaker. The combined centrifugate is then filtered through the PC filter. The PC filter is dried and weighed to obtain the weight of the residue.

#### 2.2.2 Preparation of TEM specimens

The PC filter is placed in 40 mL of filtered distilled water in a 50 mL glass beaker. The beaker is treated for 5 min in an ultrasonic bath to remove the particulate material from the filter and disperse it in the water. The PC filter is removed and discarded. Aliquots of the particulate suspension are then filtered through 25 mm diameter 0.2 μm pore size PC filters, using the filtration methods specified in Section 12 of ISO 13794 (23). These filtration methods ensure that the particulate deposits on the filters are uniform. It has been found that filtered volumes equivalent to 0.03 mL, 0.1 mL, 0.3 mL, 1.0 mL, 3.0 mL and 10 mL of the original 40 mL volume represent a sufficient range that includes satisfactory filter loadings for TEM analysis.

After drying, TEM specimens are prepared from the PC filters using the methods specified in Section 12.4 of ISO 13794 (23).

#### 2.2.3 TEM analysis

TEM specimen grids were selected for counting such that, wherever possible, there were approximately 10 fibers of the length range being counted on each grid opening. If the fiber loading of 10 fibers per grid opening could not be achieved, TEM specimen grids corresponding to a larger filtered aliquot were selected, provided that the grids did not exhibit an obscuration greater than approximately 25% of the area of each grid opening.

Fiber identification and counting were according to the procedures of Annex D and Annex E of ISO 13794 (23), with the exception that a minimum aspect ratio of 3:1 was applied for all fiber lengths ≥0.5 μm, rather than the 5:1 that is specified in ISO 13794. A magnification of approximately 21,000 was used for enumeration of amphibole fibers of lengths 0.5 μm – 5 μm, and approximately 11,000 for amphibole fibers longer than 5 μm. After recognition of the importance of fiber width in the toxicology of asbestos fibers, in later analyses in this study the widths of amphibole fibers less than approximately 5 mm as viewed on the fluorescent screen were measured at an increased magnification of approximately 60,000 to provide a more accurate measurement of width.

Where possible within reasonable analytical effort, a minimum of 100 amphibole fibers of lengths 0.5 μm–5 μm and a minimum of 100 amphibole fibers longer than 5 μm were counted. The data for each sample were processed in a custom Excel^®^ spreadsheet. Mass fractions were calculated from the amphibole fiber dimensions using a density of 3.1 g/cc and a rectangular cross-section model in which the height of a fiber is assumed to be half of the observed width (23, 24). This assumption is supported by the fact that almost all amphibole fibers appear thinner in the TEM image when the specimen grid is tilted to 45°.

## 3 Results

### 3.1 Individual mine sources

**Figure 2:**
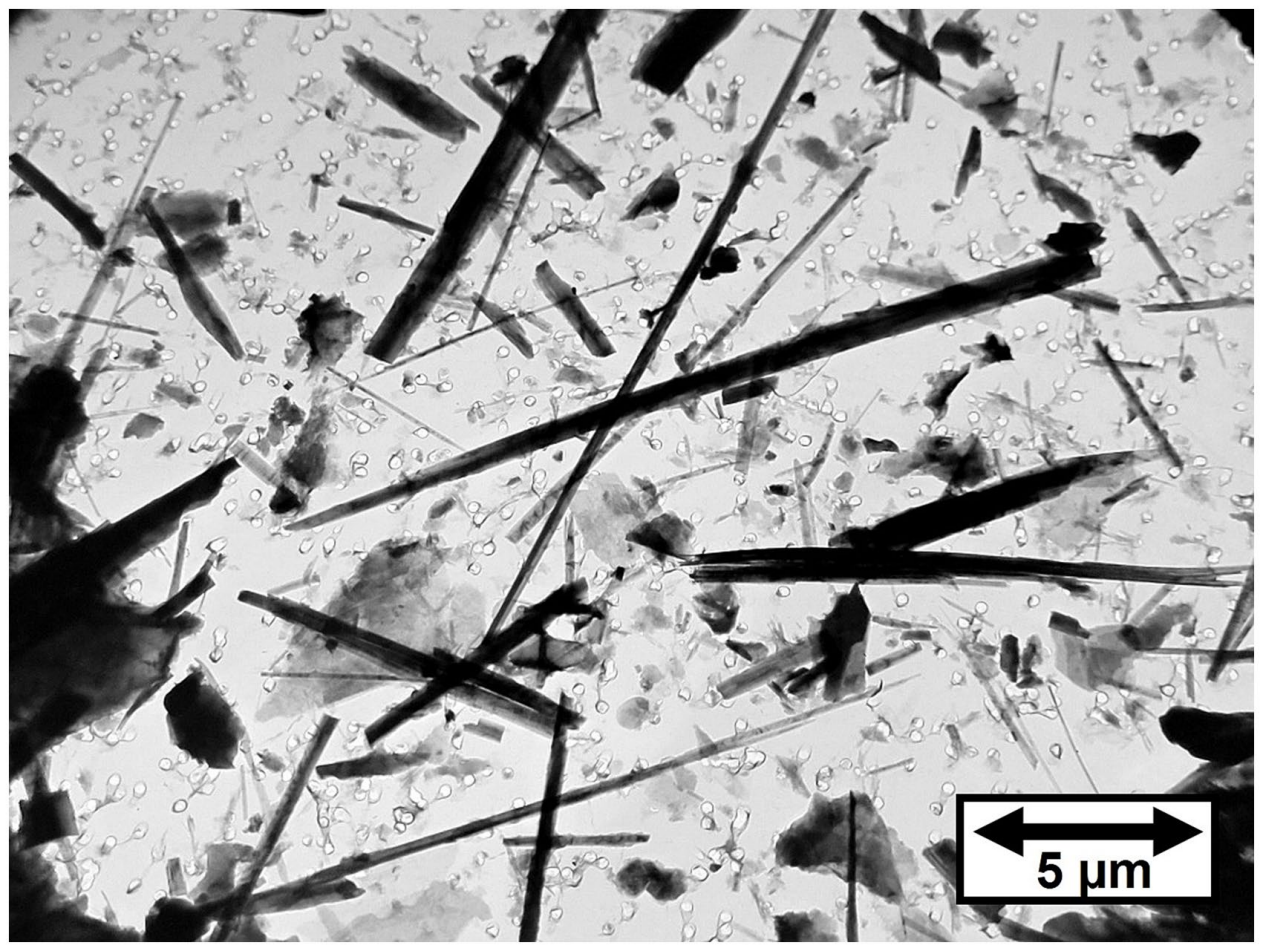
TEM micrograph of tremolite/actinolite fibers in Bell 4 T-500 chrysotile.

Figure 2 shows a TEM micrograph of tremolite/actinolite fibers in Bell 4 T-500 chrysotile. The fibers are a mixture of tremolite and actinolite with a range of iron content. It is clear that the tremolite/actinolite includes a substantial proportion of asbestiform fibers. The tremolite/actinolite mass fractions and fiber concentrations for individual mine sources are shown in Tables 1, 2. In Table 1, there are three samples (Jeffrey 4 T-3, Carey 4 T-5 and Bell 4 T-500) for which two separate sub-samples of the original chrysotile were analyzed. It can be seen that for each pair of measurements, the numerical fiber concentrations reported for the repeat measurements are quite consistent with the initial measurements. The repeat measurements of mass fraction are also consistent, although these values can sometimes be affected by the presence of a small number of disproportionately thick fibers. Chrysotile from the Bell mine stands out as having the highest mass fraction and fiber concentration of tremolite/actinolite, with one sample showing a mass fraction of more than 1% (10993.9 ppm) and 9.22 × 10^8^ fibers longer than 5 μm per gram of chrysotile. The mass fractions and fiber concentrations of tremolite/actinolite for the two Asbestos Corporation samples in Table 1 show that chrysotile with substantial tremolite/actinolite was being marketed as late as the mid 1980’s.

**Table 1:** Canadian mines: mass fractions and fiber concentrations of tremolite/actinolite per gram of chrysotile.

	Mass fraction ppm	Fibers/gram>5 μm	No. of fibers counted	Fibers/gram 0.5 μm– ≤ 5 μm	No. of fibers counted	Fibers/gram total
Chrysotile source						
Jeffrey 4 T-3 (1)	76.89	6.24 × 10^6^	168	3.08 × 10^7^	152	3.71 × 10^7^
Jeffrey 4 T-3 (2)	55.03	5.94 × 10^6^	191	1.72 × 10^7^	140	2.31 × 10^7^
Jeffrey 3 T-12	61.42	7.16 × 10^6^	100	5.35 × 10^7^	102	6.07 × 10^7^
Lac d’Amiante	78.41	5.74 × 10^6^	202	3.23 × 10^7^	173	3.80 × 10^7^
Carey 4 T-5 (1)	36.83	1.66 × 10^6^	161	5.34 × 10^6^	136	7.00 × 10^6^
Carey 4 T-5 (2)	60.26	1.70 × 10^6^	157	6.80 × 10^6^	147	8.50 × 10^6^
Bell 4 T-500 (1)	10993.90	9.22 × 10^8^	207	5.92 × 10^9^	189	6.84 × 10^9^
Bell 4 T-500 (2)	5023.18	7.17 × 10^8^	103	4.66 × 10^9^	108	5.37 × 10^9^
Bell 3F-700	2111.35	2.11 × 10^8^	101	1.69 × 10^9^	115	1.90 × 10^9^
Bell 5R-500	3809.77	4.13 × 10^8^	108	2.57 × 10^9^	112	2.98 × 10^9^
Bell 5R-600	1922.05	1.93 × 10^8^	102	1.17 × 10^9^	103	1.36 × 10^9^
Asbestos Corporation N-563-5R (1984)	4217.30	3.45 × 10^8^	107	1.92 × 10^9^	110	2.26 × 10^9^
Asbestos Corporation Group 3 (1986)	617.11	1.26 × 10^8^	102	1.17 × 10^9^	105	1.29 × 10^9^
Baie Verte, NF Advocate 25	104.54	9.22 × 10^8^	101	5.92 × 10^9^	104	6.84 × 10^9^
Cassiar A (1968)	1.91	9.63 × 10^4^	18	1.74 × 10^5^	32	2.71 × 10^5^
Cassiar A (1978)	2.94	2.03 × 10^5^	36	3.35 × 10^5^	61	5.38 × 10^5^
Cassiar AK (1981)	17.37	1.59 × 10^6^	49	2.09 × 10^6^	113	3.67 × 10^6^
Cassiar AX (1981)	9.99	5.30 × 10^5^	102	6.51 × 10^5^	119	1.18 × 10^6^
Cassiar AY (1984)	107.01	6.01 × 10^6^	188	1.77 × 10^7^	148	2.37 × 10^7^
Clinton Creek, YT	60.82	4.25 × 10^6^	101	1.31 × 10^7^	101	1.74 × 10^7^

**Table 2:** United States, Brazil and Zimbabwe mines: mass fractions and fiber concentrations of tremolite/actinolite per gram of chrysotile.

	Mass fraction ppm	Fibers/gram>5 μm	No. of fibers counted	Fibers/gram 0.5 μm– ≤ 5 μm	No. of fibers counted	Fibers/gram total
Chrysotile source						
Vermont 60	667.40	1.07 × 10^7^	100	4.89 × 10^7^	107	5.96 × 10^7^
Vermont H	58.76	1.71 × 10^6^	41	4.93 × 10^6^	96	6.63 × 10^6^
Coalinga COF-25	0.056	6.96 × 10^3^	1	6.96 × 10^3^	1	1.39 × 10^4^
Coalinga RG-144	<0.00011	<7.13 × 10^3^	0	<7.13 × 10^3^	0	<7.13 × 10^3^
Coalinga RG-244	0.010	<9.36 × 10^3^	0	9.35 × 10^3^	1	9.35 × 10^3^
Coalinga RG-244*	0.025	1.39 × 10^4^	2	<6.97 × 10^4^	0	1.39 × 10^4^
Coalinga SG-145*	0.004	<4.28 × 10^3^	0	4.28 × 10^3^	1	4.28 × 10^3^
Minaçu CB-4 T	0.11	2.04 × 10^4^	1	1.22 × 10^5^	5	1.42 × 10^5^
Minaçu CB-7TF	14.80	1.90 × 10^5^	10	6.98 × 10^5^	11	8.88 × 10^5^
Zimbabwe C&G #1	3.38	1.70 × 10^5^	10	2.23 × 10^5^	13	3.92 × 10^5^
Zimbabwe C&G 1619	1.44	5.70 × 10^4^	11	3.00 × 10^5^	19	3.57 × 10^5^

^*^2 Amosite fibers excluded – probable contamination from source in mill.

In Table 2, five measurements on four different grades of Coalinga chrysotile are reported. The residues from acid-alkali dissolution of Coalinga chrysotile are much lower than those from other sources of chrysotile, and this allows the residue from a greater weight of chrysotile to be examined in the TEM analysis. Tremolite/actinolite was detected in 3 of the 5 samples, but at mass fractions much lower than 1 ppm and close to the analytical sensitivity of the method.

The results for two grades of Minaçu, Brazil chrysotile are shown in Table 2. The tremolite/actinolite mass fraction in the primary production grade (CB-4 T) was 0.11 ppm. The other grade (CB-7TF) was a short grade material collected from the baghouse filters which contained a tremolite/actinolite mass fraction of 14.80 ppm.

The results for two grades of chrysotile from Zimbabwe in Table 2 exhibit low concentrations of tremolite/actinolite (3.38 ppm and 1.44 ppm).

### 3.2 Amosite contamination in UICC-A and UICC-B chrysotile

The results for UICC-A and UICC-B chrysotile are shown in Table 3. Three analyses were made for each of the two standards. Samples 1 and 2 were separate sub-samples taken from an original bag of each chrysotile standard. Samples “P” were sub-samples taken from a set of UICC standards provided to the author by Dr. V. Timbrell, one of the scientists who organized production and characterization of the standards.

**Table 3:** UICC-A and UICC-B chrysotile: mass fractions and fiber concentrations of tremolite/actinolite and amosite per gram of chrysotile.

	Mass fraction ppm	Fibers/gram>5 μm	No. of fibers counted	Fibers/gram 0.5 μm– ≤ 5 μm	No. of fibers counted	Fibers/gram total
Tremolite/actinolite						
UICC-A Sample 1	1.11	1.29 × 10^5^	3	1.59 × 10^5^	2	2.88 × 10^5^
UICC-A Sample 2	2.02	1.08 × 10^5^	3	9.55 × 10^5^	4	1.06 × 10^6^
UICC-A Sample P	2.87	1.99 × 10^5^	9	2.42 × 10^6^	50	2.62 × 10^6^
UICC-B Sample 1	132.08	8.61 × 10^6^	173	7.01 × 10^7^	180	7.87 × 10^7^
UICC-B Sample 2	213.08	1.39 × 10^7^	147	1.34 × 10^8^	180	1.47 × 10^8^
UICC-B Sample P	192.34	1.50 × 10^7^	184	1.12 × 10^8^	146	1.27 × 10^8^
Amosite						
UICC-A Sample 1	5.96	1.37 × 10^6^	32	2.79 × 10^6^	35	4.16 × 10^6^
UICC-A Sample 2	10.29	1.27 × 10^6^	38	4.71 × 10^6^	20	5.98 × 10^6^
UICC-A Sample P	12.61	1.16 × 10^6^	54	4.91 × 10^6^	101	6.07 × 10^6^
UICC-B Sample 1	6.76	2.99 × 10^5^	6	1.17 × 10^6^	3	1.47 × 10^6^
UICC-B Sample 2	17.79	2.83 × 10^5^	3	1.48 × 10^6^	2	1.77 × 10^6^
UICC-B Sample P	14.57	1.06 × 10^6^	13	5.37 × 10^6^	7	6.43 × 10^6^

In addition to tremolite/actinolite fibers, Amosite fibers were also detected in both UICC-A and UICC-B chrysotile. The mass fraction and fiber concentration results are shown separately for tremolite/actinolite and Amosite in Table 3. A clue as to the source of the Amosite is provided by the fact that Amosite was not detected in any of the chrysotile sources analyzed that are known or suspected to be constituents of these two standards. Amosite was not detected in either of the Zimbabwe chrysotile samples (Table 2), nor in any of the chrysotile samples from sources in Québec, at least some of which contributed chrysotile for preparation of the UICC-B standard. The mass fractions and tremolite/actinolite fiber concentrations in the Zimbabwe chrysotile samples are quite consistent with those of the UICC-A samples shown in Table 3. Moreover, the Amosite mass fractions and fiber concentrations in the UICC-A samples are consistent with the Amosite mass fractions and fiber concentrations in the UICC-B samples. This leads to the suggestion that the UICC chrysotile standards were contaminated by Amosite during production, given that a UICC Amosite standard was also produced as part of this group of standards in the same facilities.

#### 3.2.1 UICC-B chrysotile

**Figure 3:**
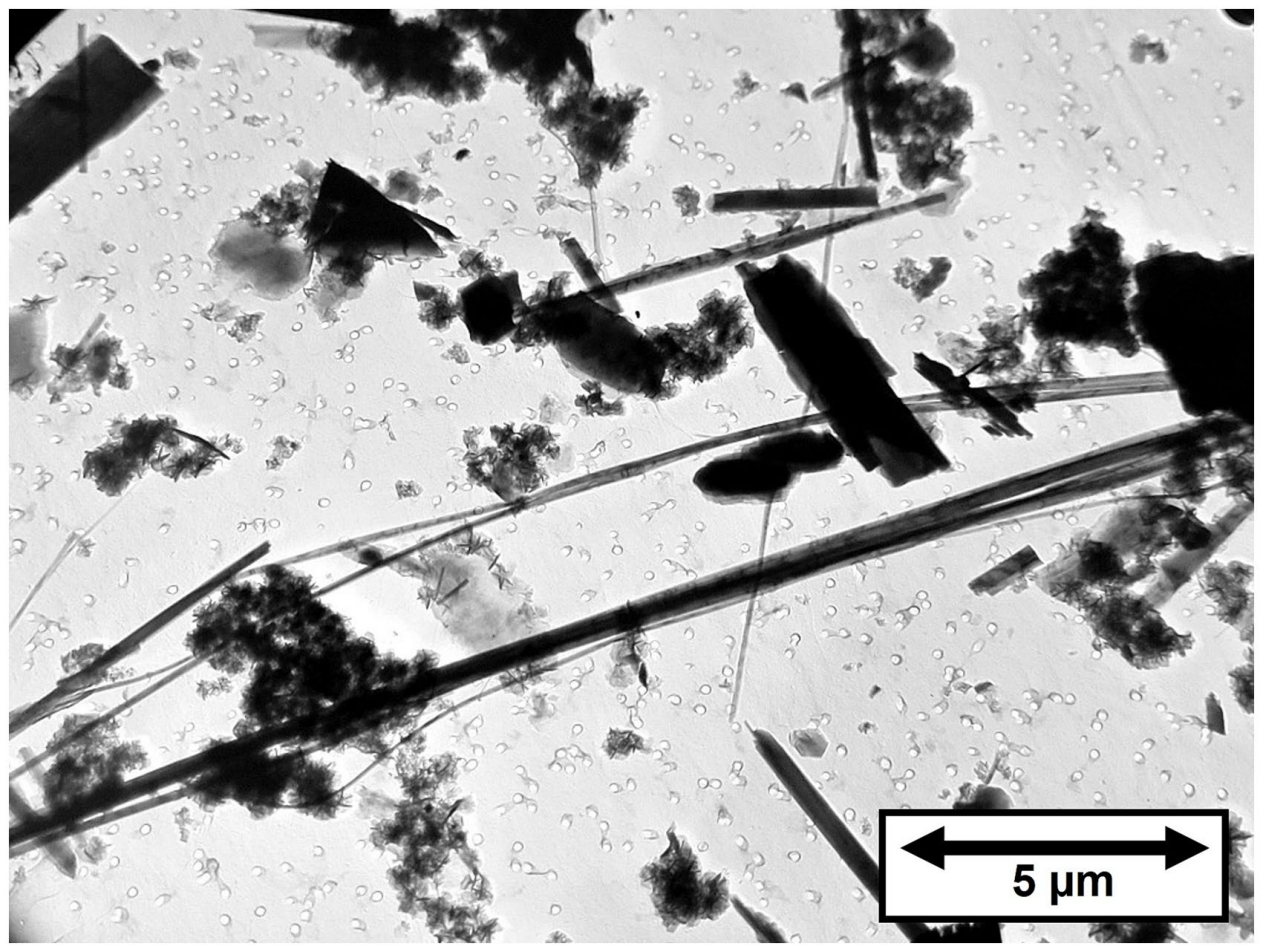
TEM micrograph of tremolite/actinolite fibers in UICC-B Canadian chrysotile.

As stated earlier, the UICC-B standard was prepared by mixing chrysotile from 8 different Québec mines in proportions intended to approximate the production volume from each mine. It is known that 50% of the chrysotile contributed to the standard was from the Jeffrey mine at Asbestos, Québec (On December 15, 2020, the town was re-named Val-des-Sources). In Table 1, the mean of the three results for Jeffrey chrysotile is 64.45 ppm or 6.45 × 10^6^ fibers/g > 5 μm in length of tremolite/actinolite. The mean of the three results for UICC-B chrysotile is 179.17 ppm or 1.25 × 10^7^ fibers/g > 5 μm in length of tremolite/actinolite, significantly higher than the corresponding values for Jeffrey chrysotile. The Bell mine was one of the major mines operating at the time the UICC standards were produced, so chrysotile from there would have been one of the contributing sources for the UICC-B standard. The 7.143% contribution from the Bell mine by itself can explain why the tremolite/actinolite content of UICC-B chrysotile is higher than any of the other possible contributing sources that were analyzed. The contribution of tremolite/actinolite from the Bell mine would amount to a range of 150.81–785.29 ppm, or 1.38 × 10^7^–6.59 × 10^7^ fibers/g > 5 μm in length, the lower ends of which are consistent with the measurements on UICC-B chrysotile. No information is available about the dates of production of the Bell samples that were analyzed, but it is clear that the tremolite/actinolite content in chrysotile from the Bell mine far exceeds those from any of the other samples analyzed. Figure 3 shows a TEM micrograph of the amphibole fibers in UICC-B chrysotile. As the data show that there are approximately 36 tremolite/actinolite fibers of all lengths for each fiber of Amosite, the majority, if not all, of the fibers in Figure 3 are tremolite/actinolite.

**Figure 4:**
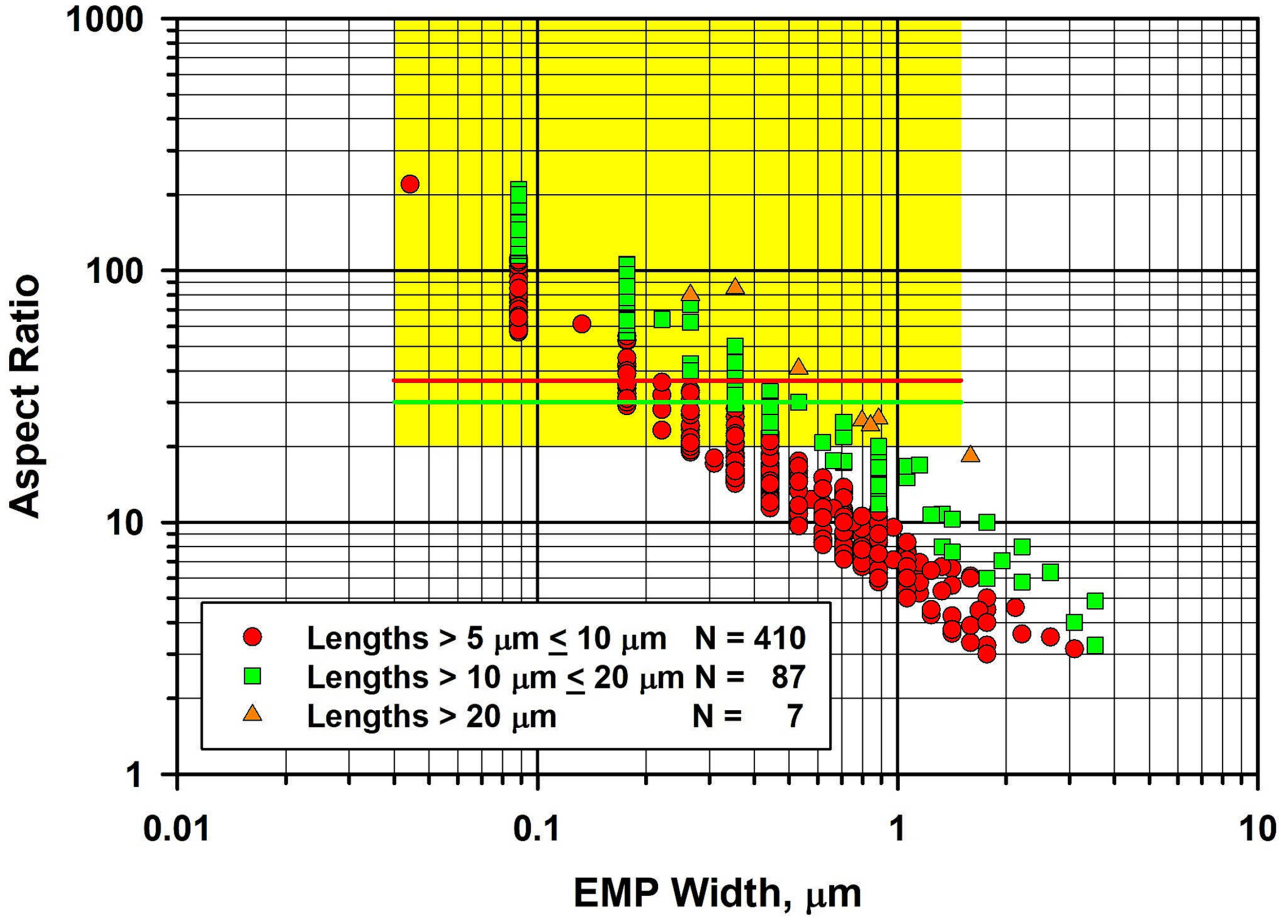
Width vs. aspect ratio plot for tremolite/actinolite fibers in UICC-B chrysotile. Combined data from 3 analyses for fibers longer than 5 μm.

In Figure 4, the fiber dimensional data for tremolite/actinolite fibers longer than 5 μm in the three samples of UICC-B chrysotile analyzed are combined. In this plot, approximately 21.4% of fibers longer than 5 μm are extra-criteria fibers (25), confirming that UICC-B chrysotile contains a substantial proportion of asbestiform tremolite/actinolite fibers.

Because UICC-B chrysotile has been the basis of so many laboratory studies, it was considered useful to present the full size distribution data, so that the possible effects on past biological studies can be evaluated. The length, width and aspect ratio distributions for the combined data from the three samples analyzed can be found in the Supplementary materials.

### 3.3 Balangero chrysotile

**Figure 5:**
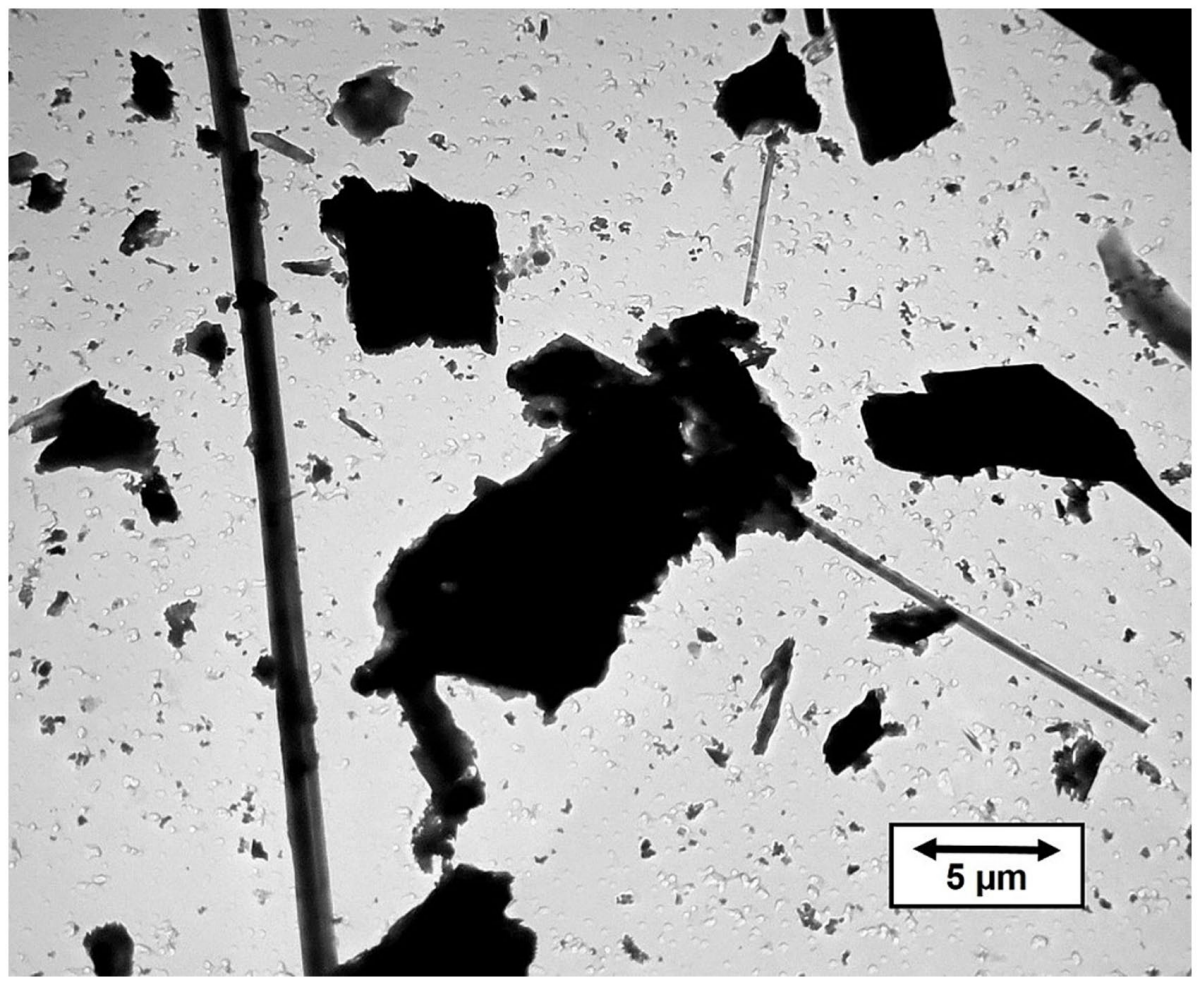
TEM micrograph of asbestiform diopside fibers in Balangero chrysotile.

**Figure 6:**
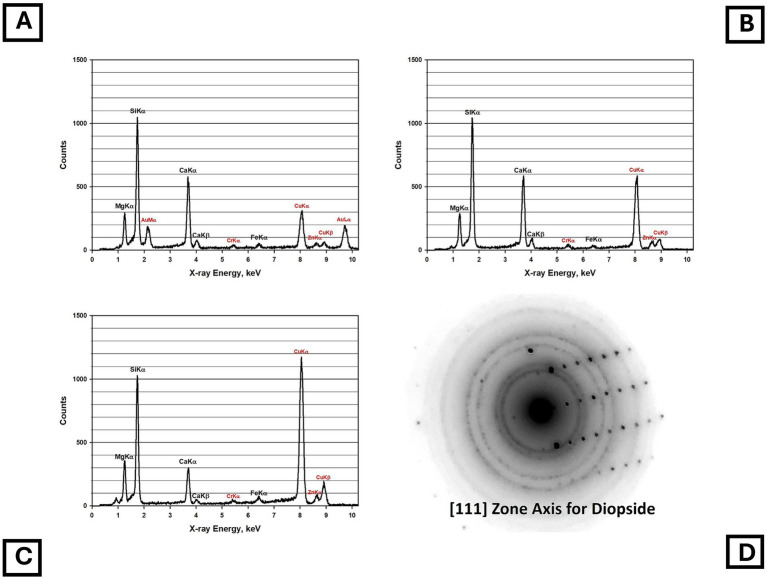
**(A)** EDXA spectrum of asbestiform diopside fiber in Balangero chrysotile. **(B)** EDXA spectrum of reference diopside. **(C)** EDXA spectrum of NIST SRM 1867 tremolite. (Peaks labeled in red in the spectra are system peaks that originate from the TEM grid and the specimen holder.) **(D)** Zone axis SAED pattern obtained from asbestiform diopside fiber in Balangero chrysotile. The pattern indexes as the [111] zone axis of diopside but is inconsistent with tremolite.

Four samples of Balangero chrysotile were analyzed. In each of the samples, an asbestiform fiber variety was present that was identified as diopside. No fibers with compositions consistent with Balangeroite (26) were detected during the TEM analyses. Figure 5 shows a TEM micrograph of typical diopside fibers found in the Balangero chrysotile samples. Figure 6A shows an EDXA spectrum obtained from a typical fiber which can be compared with the EDXA spectra from reference diopside (Figure 6B) and tremolite (Figure 6C). X-ray peaks labeled in red are system peaks that originate from the TEM grid and the specimen holder. The calcium peak in the diopside spectrum is approximately double the size of the corresponding peak in the tremolite spectrum. The zone axis selected area electron diffraction (SAED) pattern shown in Figure 6D was indexed as the [111] zone axis of diopside, but the SAED pattern is not consistent with any zone axis of tremolite. The existence of diopside in an asbestiform habit has been reported by Belluso et al. (27, 28).

**Figure 7:**
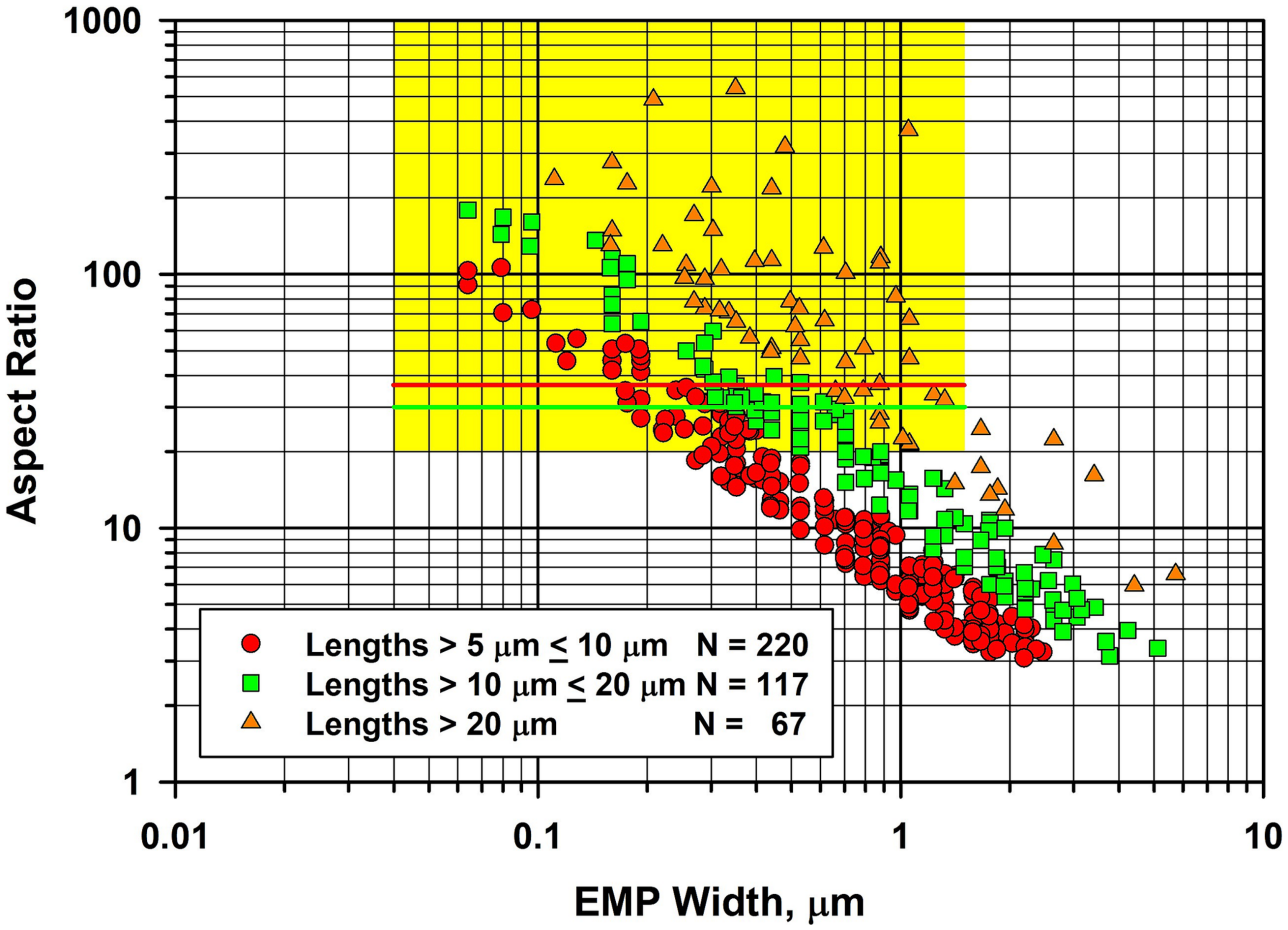
Width vs. aspect ratio plot for diopside fibers in Balangero chrysotile. Combined data from four analyses for fibers longer than 5 μm.

During the TEM examinations, most of the fibers present were diopside, along with antigorite fibers that showed evidence of chemical attack from the acid/alkali treatment. The asbestiform habit of the diopside is illustrated in Figure 7, in which there is a large proportion of thin high aspect ratio fibers. In Figure 7, the fiber dimensional data for diopside fibers longer than 5 μm are combined for the four samples of Balangero chrysotile analyzed. Some of the diopside fibers were very long and extended over several grid openings of the TEM grid. The maximum length of a diopside fiber found was in Sample 6DS3D, in which a diopside fiber 389 μm in length and 1.05 μm in width was encountered. Partially dissolved antigorite fibers were also present in the residues from the acid/alkali refluxing procedure.

Tremolite/actinolite was also detected in Balangero chrysotile, but at very low concentrations. The mass fractions and fiber concentrations for both diopside and tremolite/actinolite are shown in Table 4. The mass fractions of diopside exceed all of the tremolite/actinolite mass fractions found in other mines, with the exception of the Bell mine. The size distributions of the diopside are different from those of tremolite/actinolite in other mines in that the concentrations of fibers with lengths between 0.5 μm and 5 μm are approximately the same as for those >5 μm. For tremolite/actinolite in other mines, the concentration of the short fibers is generally about an order of magnitude greater than that for fibers >5 μm. Although tremolite/actinolite concentrations were very low, fibers of tremolite/actinolite were consistently detected in each of the samples analyzed. However, it is clear that the major consideration with respect to mineral fibers in Balangero chrysotile is diopside (26).

**Table 4:** Balangero chrysotile: mass fractions and fiber concentrations per gram of chrysotile for diopside and tremolite/actinolite.

	Mass fraction ppm	Fibers/gram>5 μm	No. of fibers counted	Fibers/gram 0.5 μm– ≤ 5 μm	No. of fibers counted	Fibers/gram total
Diopside						
Balangero SD5	1042.64	3.04 × 10^7^	100	4.17 × 10^7^	104	7.22 × 10^7^
Balangero 7D	1377.29	2.26 × 10^7^	100	2.96 × 10^7^	100	5.22 × 10^7^
Balangero 4ZX	789.06	1.78 × 10^7^	102	1.65 × 10^7^	98	3.44 × 10^7^
Balangero 6DS3D	1507.33	2.65 × 10^7^	102	2.08 × 10^7^	95	4.73 × 10^7^
Tremolite/Actinolite						
Balangero SD5	0.96	3.04 × 10^5^	1	4.01 × 10^5^	1	7.05 × 10^5^
Balangero 7D	60.45 (0.027)*	4.52 × 10^5^	2	<2.96 × 10^5^	0	4.52 × 10^5^
Balangero 4ZX	3.10	3.50 × 10^5^	2	8.43 × 10^5^	5	1.19 × 10^6^
Balangero 6DS3D	0.37	5.19 × 10^5^	2	6.58 × 10^5^	3	1.18 × 10^6^

*The mass fraction value is dominated by one fiber bundle, 24.7 μm x 2.64 μm. The value in parentheses excludes this bundle.

The composition of Balangeroite is reported as:

${\left({\mathrm{Mg,Fe}}^{2+}{,Fe}^{3+}{,Mn}^{2+}\right)}_{42}{Si}_{16}O_{54}{\left(OH\right)}_{40}$

**Figure 8:**
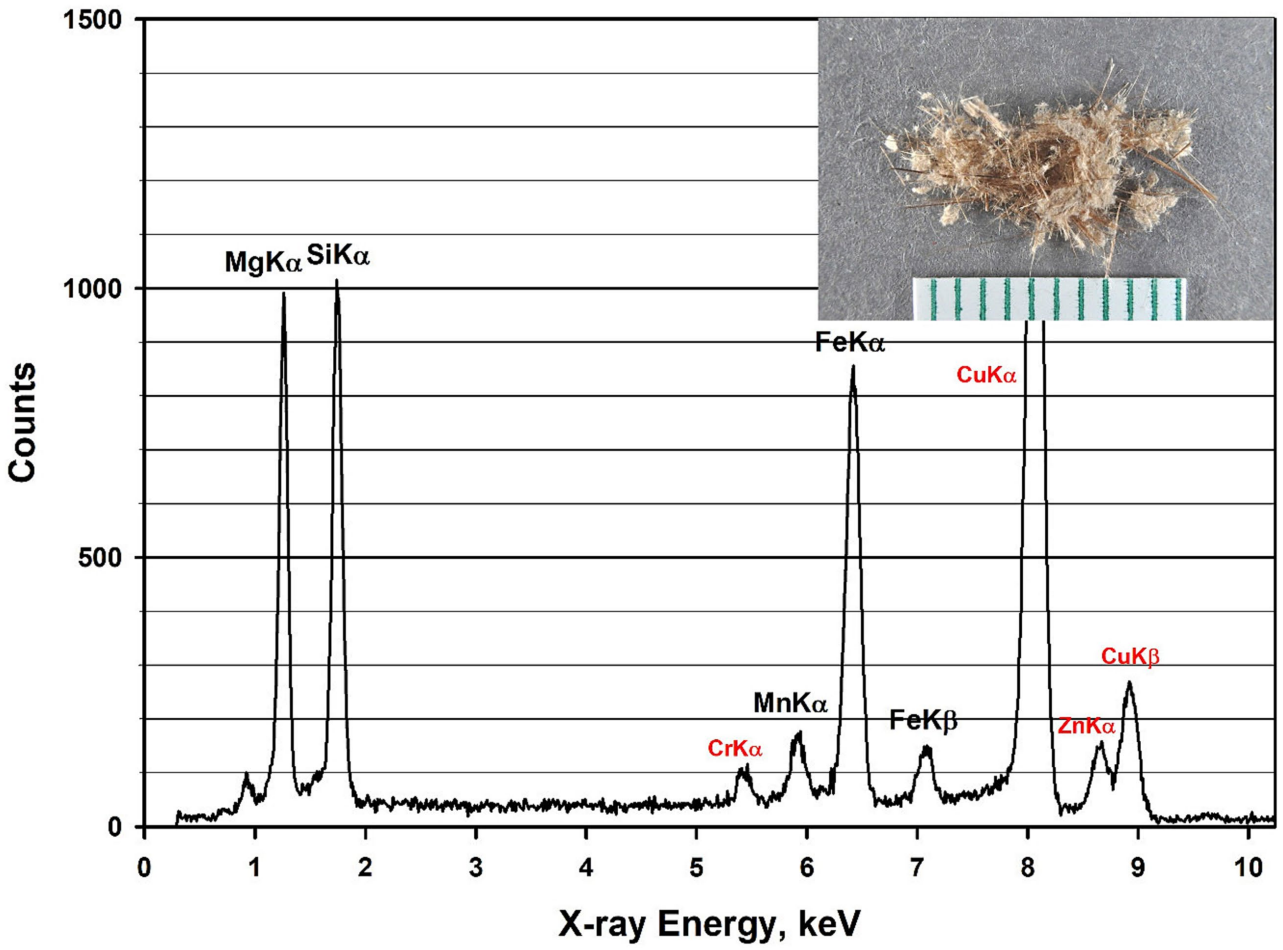
EDXA spectrum of Balangeroite. Peaks labeled in red are system peaks that originate from the TEM grid and the specimen holder. Inset photograph shows sample of Balangeroite.

An EDXA spectrum from a fiber of Balangeroite is shown in Figure 8; it exhibits a very high Mg/Si ratio with peaks from manganese and iron. The inset photograph shows a sample of Balangeroite. Since no fibers with compositions consistent with Balangeroite were detected during the analyses of the four Balangero chrysotile samples, it was suspected that the Balangeroite had dissolved during the acid/alkali treatment. Accordingly, it was decided to perform a test on Balangeroite to confirm this suspicion. A weight of 1.2 mg of the sample shown in Figure 8 was submitted to the same HCl/NaOH digestion procedure as had been used to prepare the chrysotile samples. Any residue from the digestion procedure was filtered on to a pre-weighed 0.22 μm pore size PC filter with an active area of 199 mm^2^. There was no visible residue on the filter, and any residue on the filter was below 0.0001 g, the minimum detectable for the analytical balance. No mineral fibers were detected on TEM specimens prepared from the final filter. It was concluded that Balangeroite does not survive the HCl/NaOH treatment, and that it probably has a durability comparable with that of chrysotile.

### 3.4 Exposure indices

The literature refers to several different indices that are either in use or have been proposed for determination of exposure to airborne asbestos fibers measured by TEM. These are:

(a) NIOSH PCM-equivalent fibers (29) (fibers longer than 5 μm, aspect ratio ≥3:1 and with widths >0.25 μm);
(b) ISO PCM-equivalent fibers (30) (fibers longer than 5 μm, aspect ratio ≥3:1 and with widths ≥0.2 μm– ≤ 3.0 μm);
(c) Chatfield extra-criteria fibers (25) (fibers with dimensions outside of the range exhibited by non-asbestiform amphibole cleavage fragments). These are:

(i) for fibers with lengths >5 μm– ≤ 10 μm, aspect ratios >35:1;
(ii) for fibers with lengths >10 μm– ≤ 20 μm, aspect ratios >30:1;
(iii) for fibers with lengths >20 μm, aspect ratios >20:1;

(d) Stanton fibers (31, 32) (fibers with lengths >8 μm and widths ≤0.25 μm);
(e) Berman & Crump protocol fibers (33–35) (fibers with lengths >10 μm and widths <0.5 μm);
(f) Lippmann mesothelioma fibers (36, 37) (fibers with lengths >5 μm and widths <0.1 μm);
(g) Lippmann lung cancer fibers (36, 37) (fibers with lengths >10 μm and widths >0.15 μm);
(h) Lippmann lung cancer fibers with Chatfield restriction (25) (Lippmann lung cancer fibers that also meet the definition of Chatfield extra-criteria fibers). This restriction is to exclude any Lippmann lung cancer fibers that are within the dimensional range of non-asbestiform cleavage fragments.

The numerical concentration of tremolite/actinolite fibers per gram of chrysotile has been calculated for each of the above exposure indices. Table 5 shows examples of these concentrations for each of the exposure indices for tremolite/actinolite in several of the chrysotile sources and UICC-B chrysotile. Corresponding data are also shown for diopside fibers in Balangero chrysotile. The full tabulation of fiber concentration data for each of the exposure indices can be found in the Supplementary materials.

**Table 5:** Examples of fiber concentrations for various exposure indices.

	Fibers >5 μm	NIOSH	ISO	Chatfield extra-criteria	Stanton	Berman & Crump	Lippmann mesothelioma	Lippmann lung cancer	Lippmann lung cancer with Chatfield restriction	Analytical sensitivity
Chrysotile source										
Jeffrey 4 T-3 (1)	6.24 × 10^6^ (168)	4.87 × 10^6^ (131)	4.83 × 10^6^ (130)	1.49 × 10^6^ (40)	3.72 × 10^5^ (10)	4.46 × 10^5^ (12)	4.46 × 10^5^ (12)	8.92 × 10^5^ (24)	4.83 × 10^5^ (13)	3.717 × 10^4^
Lac d’Amiante 4 T-3	5.74 × 10^6^ (202)	4.72 × 10^6^ (166)	4.72 × 10^6^ (166)	1.56 × 10^6^ (55)	2.27 × 10^5^ (8)	8.24 × 10^5^ (29)	5.40 × 10^5^ (19)	1.48 × 10^6^ (52)	8.81 × 10^5^ (31)	2.843 × 10^4^
Carey 4 T-5 (1)	1.66 × 10^6^ (161)	1.46 × 10^6^ (141)	1.44 × 10^6^ (139)	3.10 × 10^5^ (30)	5.16 × 10^4^ (5)	1.55 × 10^5^ (15)	7.23 × 10^4^ (7)	4.75 × 10^5^ (46)	1.65 × 10^5^ (16)	1.033 × 10^4^
Bell 4 T-500 (1)	9.22 × 10^8^ (207)	6.19 × 10^8^ (139)	6.10 × 10^8^ (137)	2.90 × 10^8^ (65)	8.02 × 10^7^ (18)	1.02 × 10^8^ (23)	1.69 × 10^8^ (38)	1.43 × 10^8^ (32)	7.57 × 10^7^ (17)	4.454 × 10^6^
Baie Verte Advocate 25	3.17 × 10^6^ (101)	3.02 × 10^6^ (96)	2.89 × 10^6^ (92)	1.26 × 10^5^ (4)	<3.15 × 10^4^ (0)	9.42 × 10^4^ (3)	<3.15 × 10^4^ (12)	5.65 × 10^5^ (18)	6.28 × 10^4^ (2)	3.141 × 10^4^
Cassiar AY (1984)	6.01 × 10^6^ (188)	5.63 × 10^6^ (176)	5.59 × 10^6^ (175)	2.88 × 10^5^ (9)	<3.20 × 10^4^ (0)	2.56 × 10^5^ (8)	3.20 × 10^4^ (1)	1.09 × 10^6^ (34)	2.24 × 10^5^ (7)	3.196 × 10^4^
Vermont 60	1.07 × 10^7^ (100)	1.03 × 10^7^ (96)	9.96 × 10^6^ (93)	5.35 × 10^5^ (5)	2.14 × 10^5^ (2)	2.14 × 10^5^ (2)	<1.08 × 10^5^ (0)	2.57 × 10^6^ (24)	2.14 × 10^5^ (2)	1.071 × 10^5^
Minaçu, Brazil CB-4 T	2.04 × 10^4^ (1)	2.04 × 10^4^ (1)	2.04 × 10^4^ (1)	<2.05 × 10^4^ (0)	<2.05 × 10^4^ (0)	<2.05 × 10^4^ (0)	<2.05 × 10^4^ (0)	<2.05 × 10^4^ (0)	<2.05 × 10^4^ (0)	2.040 × 10^4^
Zimbabwe C&G1619	5.70 × 10^4^ (11)	5.70 × 10^4^ (11)	5.70 × 10^4^ (11)	<5.19 × 10^3^ (0)	<5.19 × 10^3^ (0)	<5.19 × 10^3^ (0)	<5.19 × 10^3^ (0)	1.04 × 10^4^ (2)	<5.19 × 10^3^ (0)	5.183 × 10^3^
UICC-B Tremolite/Actinolite (Mean of 3 Samples)	1.17 × 10^7^ (504)	8.98 × 10^6^ (386)	8.87 × 10^6^ (381)	2.51 × 10^6^ (108)	1.00 × 10^6^ (43)	1.16 × 10^6^ (50)	1.23 × 10^6^ (53)	1.84 × 10^6^ (79)	7.68 × 10^5^ (33)	2.328 × 10^4^
Balangero Diopside (Mean of 3 Samples)	2.34 × 10^7^ (404)	2.10 × 10^7^ (363)	2.07 × 10^7^ (358)	6.31 × 10^6^ (109)	1.45 × 10^6^ (25)	3.41 × 10^6^ (59)	5.79 × 10^5^ (10)	1.02 × 10^7^ (177)	4.86 × 10^6^ (84)	5.787 × 10^4^

Values are in fibers per gram of chrysotile. Numbers in parentheses are the numbers of fiber on which the numerical concentrations are based.

If the mass concentration of airborne chrysotile is known, the values in Table 5 and those in the Supplementary materials allow the airborne numerical concentration of tremolite/actinolite and diopside fibers to be calculated for each of the above exposure indices.

## 4 Discussion

### 4.1 Tremolite/actinolite in individual mine samples

The mass fraction of tremolite/actinolite reported in Table 1 for Lac d’Amiante chrysotile (78.41 ppm) is consistent with value of 94 ppm reported by Millette et al. (11) for a sample of Black Lake chrysotile. These samples were both from the mine at Black Lake. Unfortunately, the limited data provided in the publication by Millette et al. do not permit calculation of numerical fiber concentrations.

Chrysotile from the Bell mine in Thetford Mines is notable in that the mass fraction of tremolite/actinolite in one measurement exceeds 1%. At this mass fraction, tremolite/actinolite should be detectable by polarized light microscopy (PLM). Examination of several sub-samples of Bell 4 T-500 chrysotile in a 1.605 refractive index liquid confirmed that tremolite/actinolite could be consistently detected by PLM, although quantification on the basis of PLM observation would not be possible because of the high degree of obscuration by the chrysotile in a 1.605 refractive index liquid. The high mass fraction and numerical fiber concentration of tremolite/actinolite in the Bell samples is consistent with the observations of tremolite/actinolite in lung tissues of workers at Thetford mines, compared with the lung tissues of workers in other Québec asbestos mining areas (3–7).

Samples of chrysotile from Cassiar, B.C. spanning the period from 1968 to 1984 were available. The results of the analyses (Table 1) show a progressive increase in the tremolite/actinolite content with time, although the increase may be related to the different grades.

Coalinga chrysotile is relatively pure and yields much smaller residues from the HCl/NaOH treatment than from the other types of chrysotile. Accordingly, TEM specimens with larger aliquots of the residues could be prepared, and these yielded much lower analytical sensitivities and limits of detection. The highest value for the mass fraction of tremolite/actinolite was 0.056 ppm. This is consistent with animal studies that compare the effects of UICC-B chrysotile and Jeffrey chrysotile with Coalinga chrysotile (38). UICC-B chrysotile and Jeffrey chrysotile produced fibrosis, whereas Coalinga chrysotile did not. This result could be either a consequence of different fiber lengths, differences in tremolite/actinolite content or both.

The primary production grade of chrysotile from the Minaçu mine in Brazil exhibited a 0.11 ppm mass fraction of tremolite/actinolite. The exposure indices for tremolite/actinolite in this chrysotile, shown in Table 5, with the exception of one fiber countable by PCM, are all below the limit of detection. This result is consistent with observations of lung-retained fiber content in workers whose chrysotile exposure was exclusively at the Cana Brava mine in Minaçu (39). The short grade chrysotile collected from the baghouse at Minaçu (CB-7TF) contained 14.80 ppm of tremolite/actinolite. Neither grade of Minaçu chrysotile contained Chatfield extra-criteria fibers, indicating that their dimensions were within the range exhibited by non-asbestiform amphibole.

### 4.2 Reasons for failure of Frank et al. to detect tremolite/actinolite in UICC-B chrysotile

A publication by Frank et al. (15) reported that no tremolite fibers were detected in a TEM count of 10,000 chrysotile fibers of UICC-B chrysotile. In an additional publication, Frank et al. reported that no tremolite fibers were detected in an increased TEM count of 20,000 fibers of UICC-B chrysotile (16). No minimum fiber length for the TEM analyses was specified, but it is generally accepted that 0.5 μm is the minimum length for reliable detection and identification in TEM analysis for mineral fibers (37, 40).

To explain the apparent discrepancy between the claims of Frank et al. and the concentrations of tremolite/actinolite fibers reported in Table 3 of this current study, it is necessary to determine the number of chrysotile fibers ≥0.5 μm in water-dispersed UICC-B chrysotile per gram of chrysotile. In 1989, there was interest in the concentrations of asbestos fibers in potable water, and an analytical method was developed to make these measurements (41). Various agencies, including the United States Environmental Agency (EPA), were commissioning such measurements and the need arose for quality assurance samples to evaluate the performance of laboratories that were performing these analyses. As part of development of an analytical method for determination of asbestos in potable water (42), the author developed a method by which sealed glass ampoules of stable suspensions of chrysotile asbestos in water could be prepared. EPA and the author collaborated in the preparation of several thousand ampoules containing five different concentrations of UICC-B chrysotile in aqueous suspension (43). All the fiber suspensions were derived by dilution of a single suspension in which a known weight of UICC-B chrysotile had been dispersed. The numerical fiber concentrations per gram of UICC-B chrysotile in these suspensions were determined by TEM analysis according to the EPA Analytical Method for Determination of Asbestos in Water (41). The results of the analyses of one ampoule for each of the five concentrations, expressed as chrysotile fibers ≥0.5 μm per gram of UICC-B chrysotile, are shown in Table 6. The mean value is 1.21 × 10^13^ fibers/g. Table 6 also shows the concentration of tremolite/actinolite fibers ≥0.5 μm per gram of UICC-B chrysotile for each of the three UICC-B chrysotile samples analyzed in this study. The mean value is 1.18 × 10^8^ fibers/g. The chrysotile/tremolite numerical ratio is 102,500. Therefore, in an analysis of 20,000 chrysotile fibers, there was only a 1 in 5 chance that a single tremolite fiber ≥0.5 μm would have been detected by Frank et al.

**Table 6:** UICC-B chrysotile: TEM measurements of the number of chrysotile fibers and tremolite/actinolite fibers per gram of chrysotile.

	Chrysotile fibers in UICC-B chrysotile fibers/g ≥ 0.5 μm	Tremolite/actinolite fibers in UICC-B chrysotile fibers/g ≥ 0.5 μm
Sample		
UICC-B chrysotile	1.22 × 10^13^	7.87 × 10^7^
	1.43 × 10^13^	1.47 × 10^8^
	1.05 × 10^13^	1.27 × 10^8^
	1.04 × 10^13^	–
	1.30 × 10^13^	–
Mean concentration	1.21 × 10^13^	1.18 × 10^8^

### 4.3 Other reports involving limit of detection issues

Egilman and Menéndez (18) reported on a case of occupational peritoneal mesothelioma in Québec, Canada from exposure to what was described in the publication as “tremolite-free chrysotile.” The chrysotile in question was from the Carey mine, which had been analyzed for the presence of tremolite by Gunter et al. (44). Although Egilman and Menéndez described the Gunter et al. result as “tremolite-free chrysotile,” in reality no measurement method is capable of such a result; all analytical methods are subject to limits of detection, nor was such a claim made by Gunter et al.

**Figure 9:**
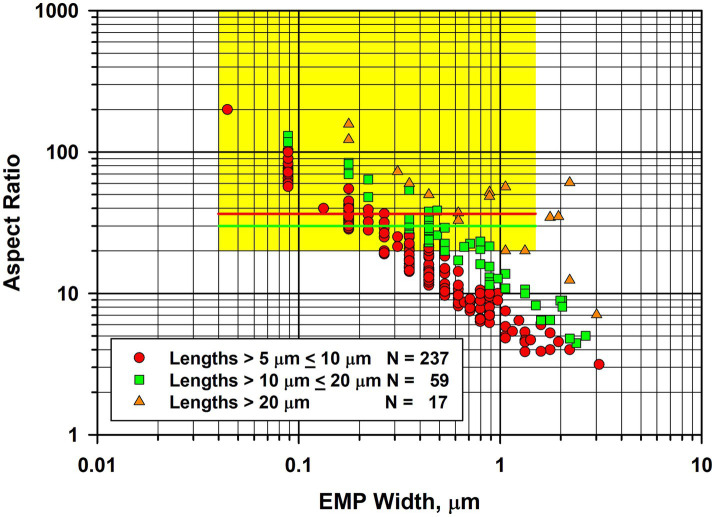
Width vs. aspect ratio plot for tremolite/actinolite fibers in Carey chrysotile. Combined data from two analyses for fibers longer than 5 μm.

The precise result published by Gunter et al. was that detection limits for their method were at least 500 ppm (i.e., 0.05%) and possibly as low as 100 ppm. Of the 10 samples analyzed, one was found to contain amphibole above the 100 ppm detection limit of their methods. The standardized SEM–EDS approach used showed approximately half of the amphiboles in this sample to be anthophyllite and the other half to be actinolite, with no confirmed tremolite. On the basis of calibrated x-ray diffraction methods, this sample contained between 500 to 1,000 ppm amphibole. Based on calcium content as a proxy for tremolite, it could contain no more than 0.2% (i.e., 2000 ppm) tremolite, although the result from this latter method was considered almost certainly to be an overestimate. Thus the Gunter et al. publication reported a limit of detection no lower than 100 ppm for tremolite. It was reported that morphology observations with the SEM and PLM did not reveal any clearly asbestiform amphiboles, but that some of the particles analyzed could be morphologically asbestiform. However, Gunter et al. indicated that it was difficult to tell at the magnification and image resolution in use. The fiber width vs. aspect ratio plot shown in Figure 9 for tremolite/actinolite fibers measured by TEM in the current work indicates that approximately 17.6% of the fibers longer than 5 μm are extra-criteria fibers that are clearly asbestiform. Accordingly, chrysotile from the Carey mine cannot be described as “tremolite-free,” thus calling into question the conclusion of Egilman and Menéndez.

Gunter et al. reported a limit of detection of 500 ppm, but possibly as low as 100 ppm. This limit of detection is quite consistent with the findings of this study, in which two samples of Carey chrysotile yielded tremolite/actinolite mass fractions of 36.83 ppm and 60.26 ppm.

### 4.4 Balangero chrysotile

There have been a number of investigations into the possible health effects of Balangeroite (45–51). There have also been two studies to assess the durability of Balangeroite in simulated lung fluids (46, 48). Groppo et al. (46) concluded that Balangeroite was more durable than crocidolite. Later experiments by Turci et al. showed that release of metal ions occurred from Balangeroite, but that tremolite was unaffected in the simulated lung fluids used. It was concluded that Balangeroite could not be considered solely responsible for the mesotheliomas observed at Balangero. In the current work, it was found that Balangeroite did not survive the HCl/NaOH treatment, and it is likely that the durability of Balangeroite is comparable to that of chrysotile.

Mirabelli et al. (47) concluded that their observations of mesothelioma cases among workers and others who were exposed to Balangero chrysotile provides evidence that tremolite-free chrysotile is carcinogenic. Ferrante et al. (50) also concluded that their data confirmed the carcinogenicity of chrysotile, in particular for pleural mesothelioma. However, although the current work shows that the mass fraction and fiber concentration of tremolite in Balangero chrysotile are very low, much higher mass fractions and fiber concentrations of asbestiform diopside fibers were found in Balangero chrysotile. The mass fractions and fiber concentrations of diopside were significantly higher than the tremolite/actinolite concentrations in many of the chrysotile samples from other sources that were examined, but lower than those in the Bell chrysotile samples. The fiber concentrations for each of the exposure indices for diopside in Balangero chrysotile shown in Table 5 and the size distribution of the diopside fibers longer than 5 μm, shown in Figure 7, strongly support the hypothesis that diopside fibers may contribute to the mesothelioma incidence at Balangero. Unlike Balangeroite, the diopside fibers are durable even in boiling 2 N hydrochloric acid. Tremolite/actinolite was also present in all four samples of Balangero chrysotile that were analyzed, but at concentrations close to the limit of detection for the measurement.

## 5 Conclusion

It appears that “tremolite-free Canadian chrysotile” does not exist. All of the Canadian sources analyzed contain tremolite/actinolite, a proportion of which is asbestiform in morphology. For the Québec chrysotile samples analyzed, the lowest mass fraction was 36.83 ppm (Carey) and the highest 10993.90 ppm (Bell).

Regardless of publications to the contrary, the UICC-B Canadian chrysotile reference standard contains substantial concentrations of tremolite/actinolite asbestos. The UICC-A and UICC-B reference chrysotile standards are also both contaminated with Amosite. Conclusions drawn in publications that refer to chrysotile from the Carey mine as “tremolite free” also have no scientific basis.

The Cana Brava mine at Minaçu, Brazil, and the mine at Coalinga, California, appear to be two sources of chrysotile in which tremolite/actinolite in the primary grades is substantially lower than 1 part per million.

The pyroxene diopside occurs in an asbestiform habit in chrysotile from Balangero, Italy. The size distribution of the diopside exhibits a greater proportion of long fibers than the tremolite/actinolite in other sources of chrysotile. Balangero chrysotile contains only traces of tremolite/actinolite (~ 1 ppm). Balangeroite is not durable and dissolves in boiling 2 M hydrochloric acid.

Given the numerous biological experiments carried out historically using UICC-B and UICC-A, it would appear that the presence and concentration of tremolite/actinolite in these two standards, and also the presence of Amosite in both standards, could raise questions about the validity or interpretation of the historical results. As one example, the inhalation experiments on rats carried out by Wagner et al. (52) showed zero mesotheliomas for UICC-A chrysotile, compared with 4 mesotheliomas for UICC-B chrysotile, a difference that could be a consequence of the much higher tremolite/actinolite content of UICC-B chrysotile.

The fiber concentrations for Bell chrysotile (Table 1) far exceed those for the other sources of Québec chrysotile that were analyzed. If possible, further review of the epidemiology could produce firm evidence for differences between former employees of the Bell mine vs. employees from other Canadian mines.

In view of the low tremolite/actinolite concentrations in chrysotile from the Cana Brava mine at Minaçu, Brazil, follow-up of the employees could provide further information on the toxicity of chrysotile.

Analyses of lung tissue from former employees of the Balangero mine in Italy (53) could provide information on the toxicity of asbestiform diopside, in view of the high fiber concentrations of asbestiform diopside in Balangero chrysotile (Table 3).

## Data Availability

The raw data supporting the conclusions of this article will be made available by the authors, without undue reservation.
