# Supplementary material for: Associated minerals in chrysotile deposits and their potential health risks
Source: Front Public Health. 2025 May 7;13:1583469. doi: 10.3389/fpubh.2025.1583469 (PMC12092358; doi:10.3389/fpubh.2025.1583469)
Supplement: Supplementary file 1 [file Data_Sheet_1.DOCX]

Supplementary Material

# Supplementary Data

**1.1 Size distribution of tremolite/actinolite fibers in UICC‑B chrysotile.**


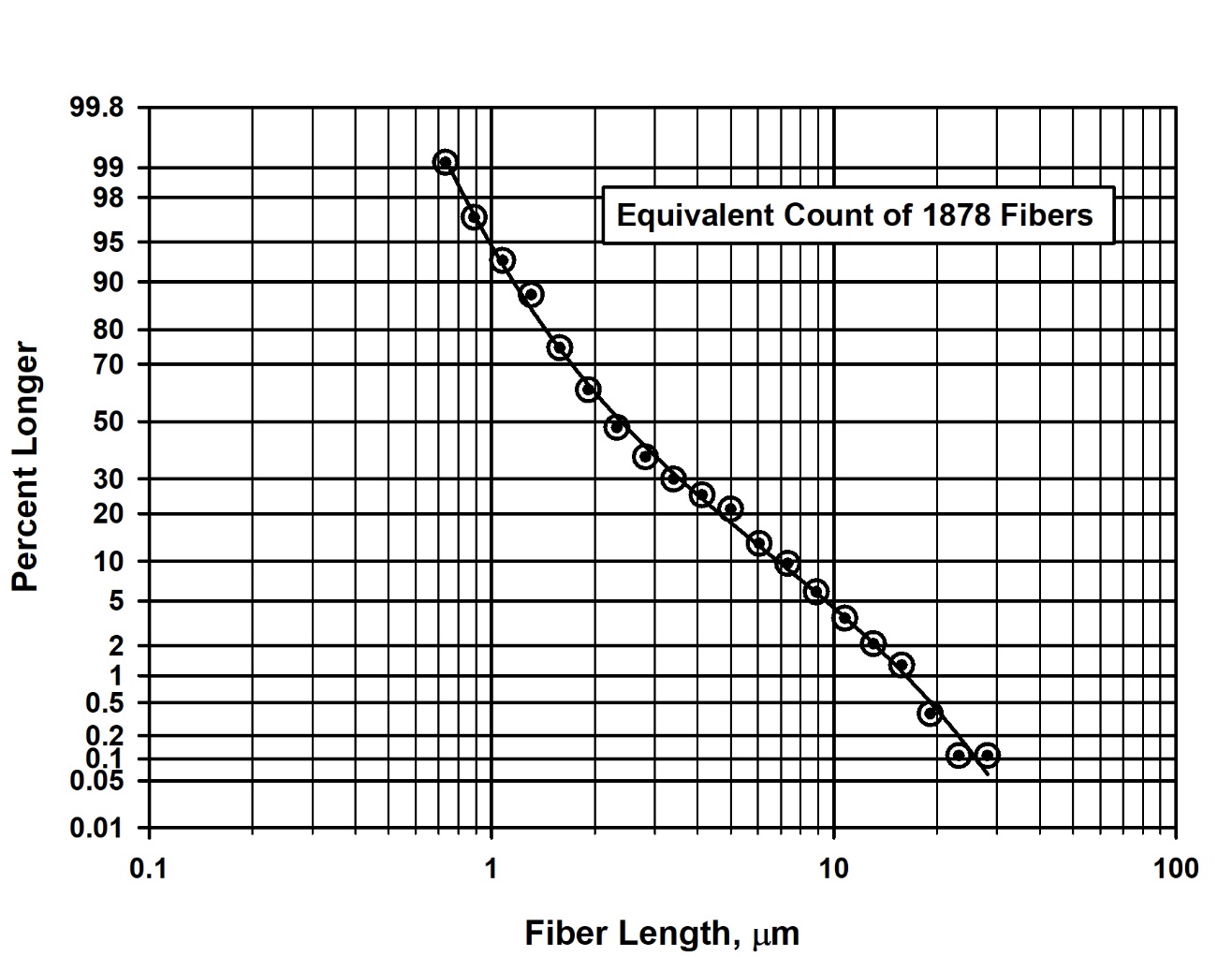
In view of the importance of the UICC chrysotile standards in past biological research, the size distributions for tremolite/actinolite and Amosite for the full range of fiber length are presented below. The size distributions were produced by combining separate fiber counts for fibers >5 µm and fibers 0.5 µm – 5 µm.

Figure S1. Length distribution of tremolite/actinolite fibers in UICC-B chrysotile.


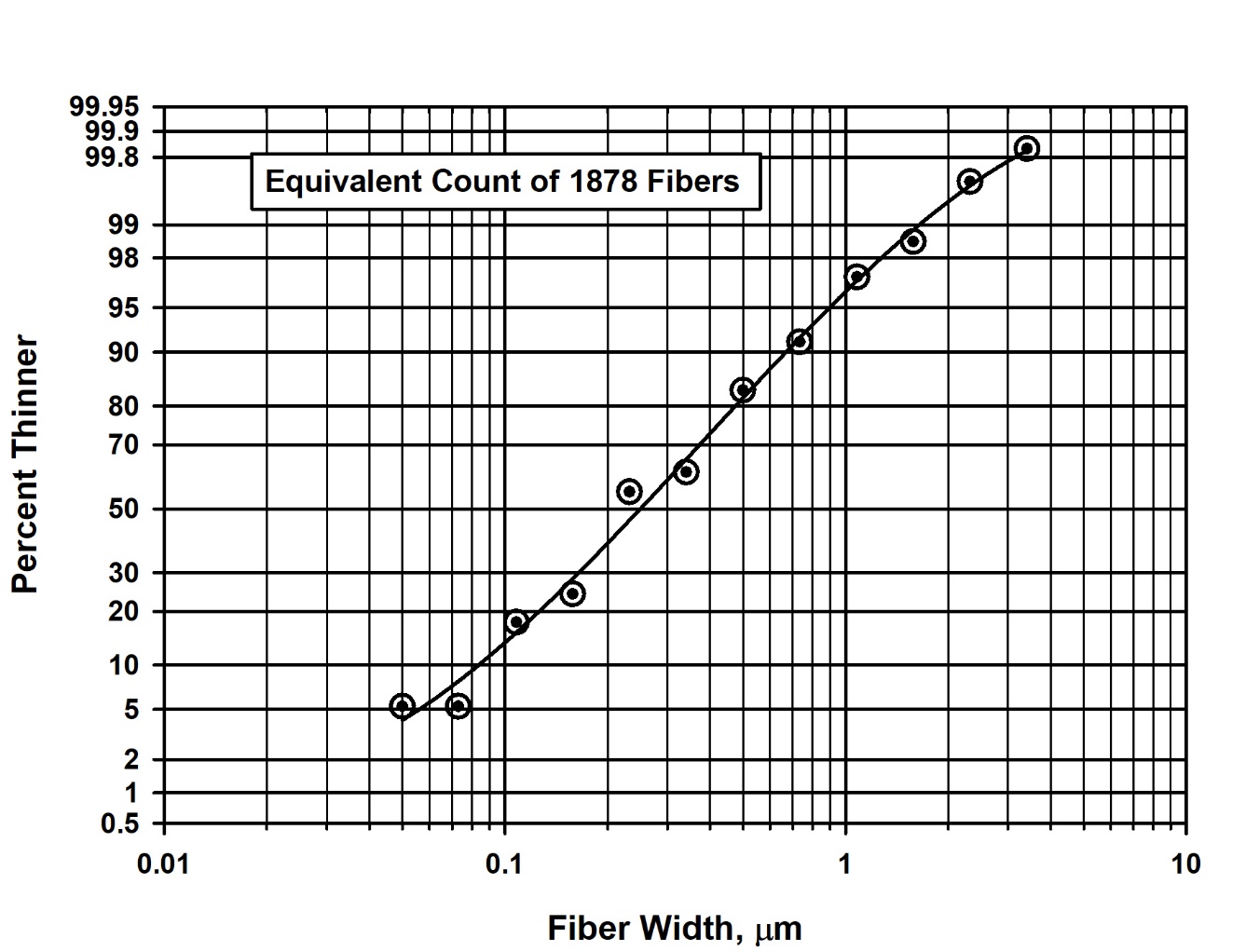


Figure S2. Width distribution of tremolite/actinolite fibers in UICC-B chrysotile.


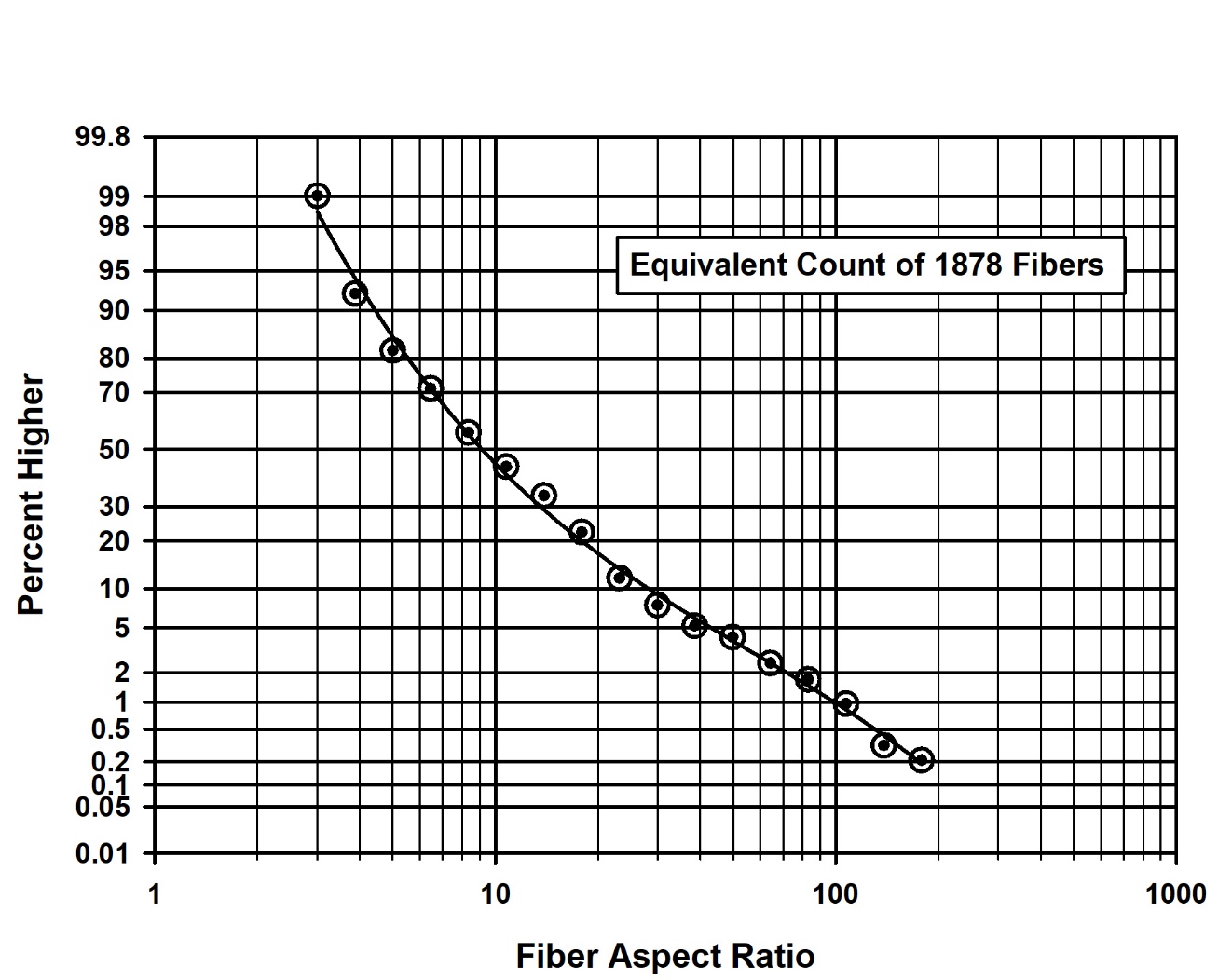


Figure S3. Aspect ratio distribution of tremolite/actinolite fibers in UICC-B chrysotile.

**1.2 Size distribution of Amosite fibers in UICC‑A chrysotile.**


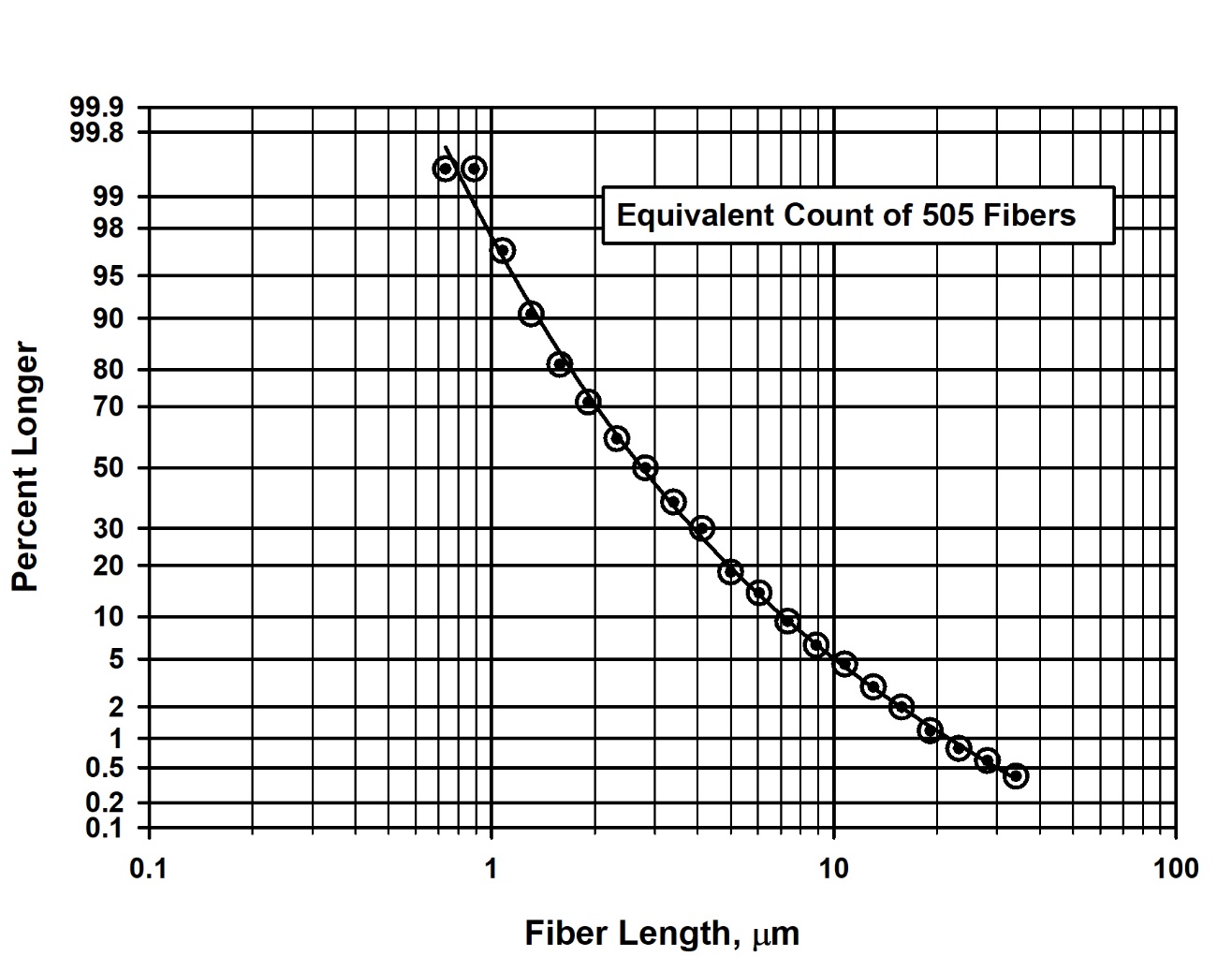


Figure S4. Length distribution of Amosite fibers in UICC-A chrysotile.


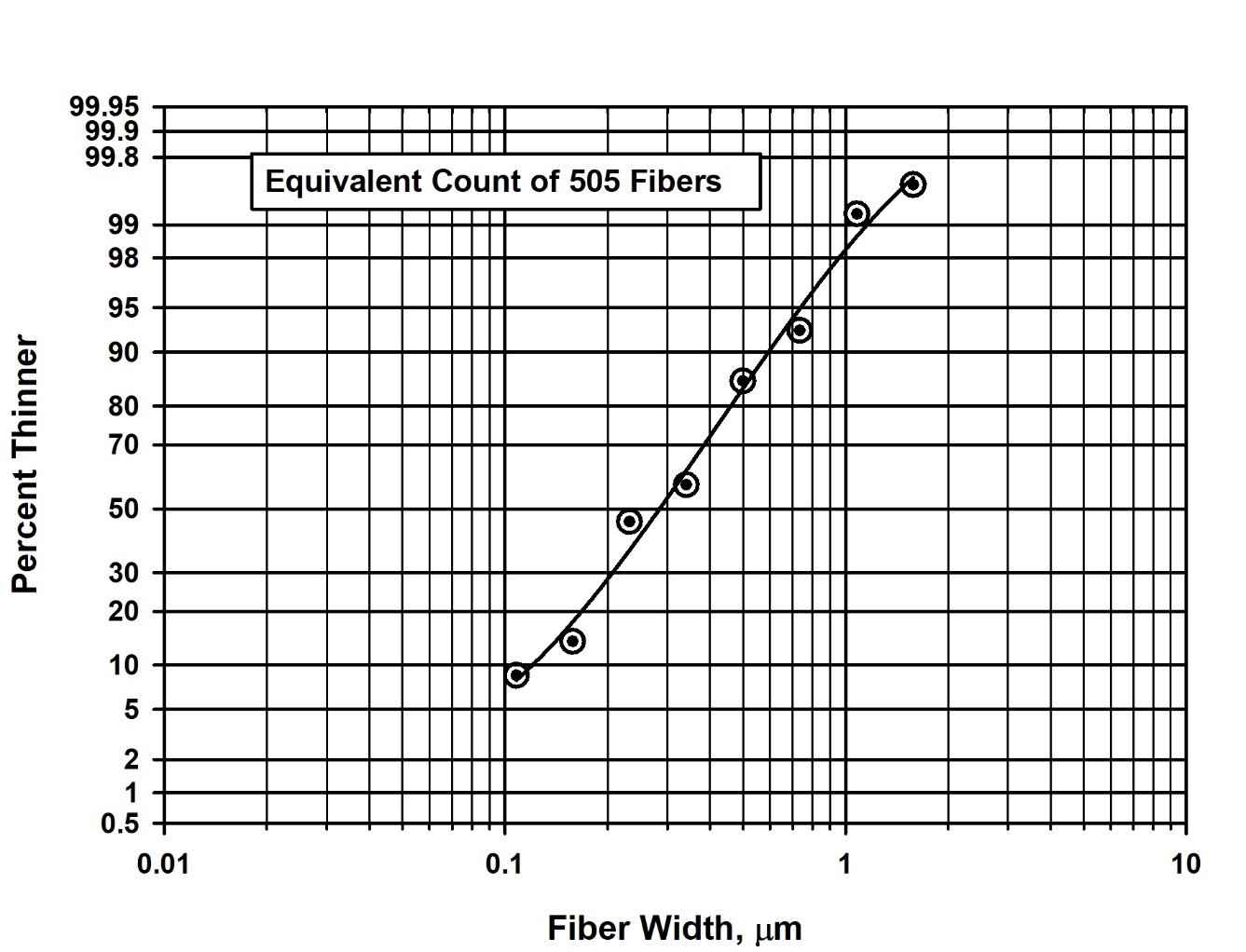


Figure S5. Width distribution of Amosite fibers in UICC-A chrysotile.


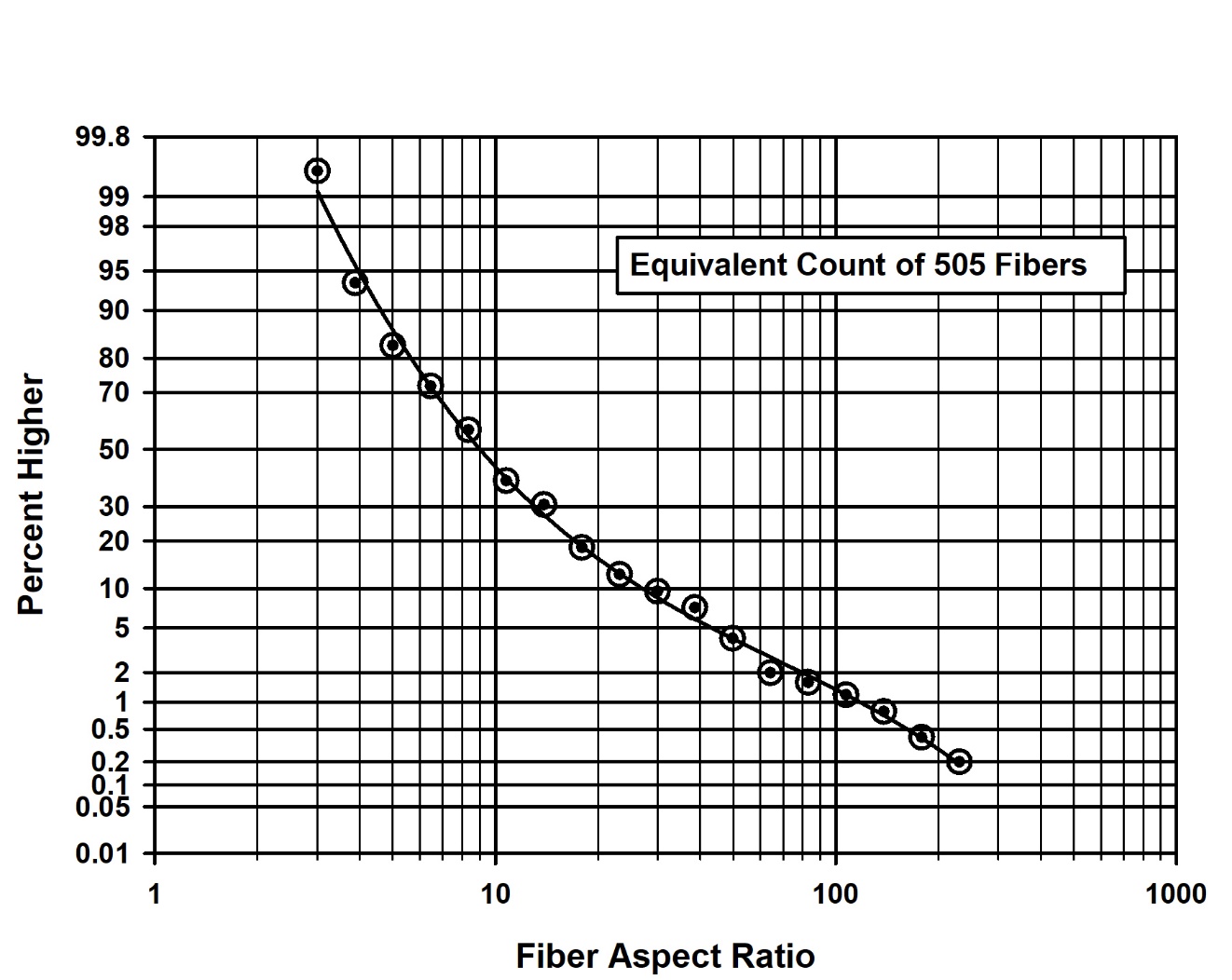


Figure S6. Aspect ratio distribution of Amosite fibers in UICC-A chrysotile.

**1.3 Fiber concentrations for various exposure indices for mineral fibers.**

If the mass concentration of airborne chrysotile is known, the values in the following Tables S1-S6 allow the airborne numerical concentration of tremolite/actinolite or diopside fibers to be calculated for each of the specified exposure indices.

Table S1. Québec Chrysotile: Fiber concentrations for various exposure indices for tremolite/actinolite fibers. Values are in fibers per gram of chrysotile. Numbers in parentheses are the numbers of fibers on which the numerical concentrations are based.

|  | Fibers  >5 µm | NIOSH | ISO | Chatfield  Extra- Criteria | Stanton | Berman  &  Crump | Lippmann Mesothelioma | Lippmann  Lung Cancer | Lippmann Lung Cancer with Chatfield Restriction | Analytical Sensitivity |
| --- | --- | --- | --- | --- | --- | --- | --- | --- | --- | --- |
| Chrysotile Source | | | | | | | | | | |
| Jeffrey 4T-3 (1) | 6.24 x 10^6^  (168) | 4.87 x 10^6^  (131) | 4.83 x 10^6^  (130) | 1.49 x 10^6^  (40) | 3.72 x 10^5^  (10) | 4.46 x 10^5^  (12) | 4.46 x 10^5^  (12) | 8.92 x 10^5^  (24) | 4.83 x 10^5^  (13) | 3.717 x 10^4^ |
| Jeffrey 4T-3 (2) | 5.94 x 10^6^  (191) | 4.70 x 10^6^  (151) | 4.70 x 10^6^  (151) | 1.18 x 10^6^  (38) | 2.49 x 10^5^  (8) | 5.60 x 10^5^  (18) | 3.42 x 10^5^  (11) | 1.31 x 10^6^  (42) | 4.05 x 10^5^  (13) | 3.112 x 10^4^ |
| Jeffrey 3T-12 | 7.16 x 10^6^  (100) | 4.80 x 10^6^  (67) | 5.22 x 10^6^  (73) | 2.93 x 10^6^  (41) | 7.87 x 10^5^  (11) | 7.87 x 10^5^  (11) | 2.15 x 10^5^  (3) | 1.22 x 10^6^  (17) | 8.59 x 10^5^  (12) | 7.157 x 10^4^ |
| Lac d’Amiante 4T-3 | 5.74 x 10^6^  (202) | 4.72 x 10^6^  (166) | 4.72 x 10^6^  (166) | 1.56 x 10^6^  (55) | 2.27 x 10^5^  (8) | 8.24 x 10^5^  (29) | 5.40 x 10^5^  (19) | 1.48 x 10^6^  (52) | 8.81 x 10^5^  (31) | 2.843 x 10^4^ |
| Carey 4T-5 (1) | 1.66 x 10^6^  (161) | 1.46 x 10^6^  (141) | 1.44 x 10^6^  (139) | 3.10 x 10^5^  (30) | 5.16 x 10^4^  (5) | 1.55 x 10^5^  (15) | 7.23 x 10^4^  (7) | 4.75 x 10^5^  (46) | 1.65 x 10^5^  (16) | 1.033 x 10^4^ |
| Carey 4T-5 (2) | 1.70 x 10^6^  (157) | 1.22 x 10^6^  (113) | 1.20 x 10^6^  (111) | 4.11 x 10^5^  (38) | 9.73 x 10^4^  (9) | 1.08 x 10^5^  (10) | 1.62 x 10^5^  (15) | 3.03 x 10^5^  (28) | 1.08 x 10^5^  (10) | 1.081 x 10^4^ |
| Bell 4T-500 (1) | 9.22 x 10^8^  (207) | 6.19 x 10^8^  (139) | 6.10 x 10^8^  (137) | 2.90 x 10^8^  (65) | 8.02 x 10^7^  (18) | 1.02 x 10^8^  (23) | 1.69 x 10^8^  (38) | 1.43 x 10^8^  (32) | 7.57 x 10^7^  (17) | 4.454 x 10^6^ |
| Bell 4T-500 (2) | 7.17 x 10^8^  (103) | 4.94 x 10^8^  (71) | 5.64 x 10^8^  (81) | 2.93 x 10^8^  (42) | 6.96 x 10^7^  (10) | 1.18 x 10^8^  (17) | 3.48 x 10^7^  (5) | 1.46 x 10^8^  (21) | 9.75 x 10^7^  (14) | 6.964 x 10^6^ |
| Bell 3F-700 | 2.11 x 10^8^  (101) | 1.47 x 10^8^  (70) | 1.63 x 10^8^  (78) | 8.17 x 10^7^  (39) | 8.37 x 10^6^  (4) | 1.88 x 10^7^  (9) | 1.26 x 10^7^  (6) | 3.14 x 10^7^  (15) | 2.09 x 10^7^  (10) | 2.094 x 10^6^ |
| Bell 5R-500 | 4.13 x 10^8^  (108) | 3.02 x 10^8^  (79) | 3.36 x 10^8^  (88) | 1.68 x 10^8^  (44) | 2.67 x 10^7^  (7) | 5.73 x 10^7^  (15) | 3.82 x 10^6^  (1) | 8.79 x 10^7^  (23) | 7.64 x 10^7^  (20) | 3.820 x 10^6^ |
| Bell 5R-600 | 1.93 x 10^8^  (102) | 1.34 x 10^8^  (71) | 1.47 x 10^8^  (78) | 7.55 x 10^7^  (40) | 1.51 x 10^7^  (8) | 1.70 x 10^7^  (9) | 3.78 x 10^6^  (2) | 3.59 x 10^7^  (19) | 2.45 x 10^7^  (13) | 1.888 x 10^6^ |
| Asbestos Corporation  N-563-5R (1984) | 3.45 x 10^8^  (107) | 2.42 x 10^8^  (75) | 2.55 x 10^8^  (79) | 1.26 x 10^8^  (39) | 3.87 x 10^7^  (12) | 4.52 x 10^7^  (14) | 9.68 x 10^6^  (3) | 7.74 x 10^7^  (24) | 4.52 x 10^7^  (14) | 3.225 x 10^6^ |
| Asbestos Corporation Group 3 (1986) | 1.26 x 10^8^  (102) | 7.04 x 10^7^  (57) | 7.78 x 10^7^  (63) | 6.92 x 10^7^  (56) | 1.85 x 10^7^  (15) | 1.98 x 10^7^  (16) | 9.88 x 10^6^  (8) | 2.47 x 10^7^  (20) | 2.10 x 10^7^  (17) | 1.235 x 10^6^ |

Table S2. Newfoundland, British Columbia and Yukon Mines: Fiber concentrations for various exposure indices for tremolite/actinolite fibers. Values are in fibers per gram of chrysotile. Numbers in parentheses are the numbers of fibers on which the numerical concentrations are based.

|  | Fibers  >5 µm | NIOSH | ISO | Chatfield Extra- Criteria | Stanton | Berman  &  Crump | Lippmann Mesothelioma | Lippmann  Lung Cancer | Lippmann Lung Cancer with Chatfield Restriction | Analytical Sensitivity |
| --- | --- | --- | --- | --- | --- | --- | --- | --- | --- | --- |
| Chrysotile Source | | | | | | | | | | |
| Baie Verte  Advocate 25 | 3.17 x 10^6^  (101) | 3.02 x 10^6^  (96) | 2.89 x 10^6^  (92) | 1.26 x 10^5^  (4) | <3.15 x 10^4^  (0) | 9.42 x 10^4^  (3) | <3.15 x 10^4^  (12) | 5.65 x 10^5^  (18) | 6.28 x 10^4^  (2) | 3.141 x 10^4^ |
| Cassiar A (1968) | 9.63 x 10^4^  (18) | 9.63 x 10^4^  (18) | 9.63 x 10^4^  (18) | <5.35 x 10^3^  (0) | <5.35 x 10^3^  (0) | <5.35 x 10^3^  (0) | <5.35 x 10^3^  (0) | 1.60 x 10^4^  (3) | <5.35 x 10^3^  (0) | 5.348 x 10^3^ |
| Cassiar A (1978) | 2.03 x 10^5^  (36) | 1.91 x 10^5^  (34) | 1.91 x 10^5^  (34) | 1.69 x 10^4^  (3) | <5.63 x 10^3^  (0) | 5.63 x 10^3^  (1) | <5.63 x 10^3^  (0) | 4.50 x 10^4^  (8) | 1.13 x 10^4^  (2) | 5.630 x 10^3^ |
| Cassiar AK (1981) | 1.59 x 10^6^  (49) | 1.46 x 10^6^  (45) | 1.46 x 10^6^  (45) | 2.26 x 10^5^  (7) | 3.24 x 10^4^  (1) | 1.29 x 10^5^  (4) | <3.24 x 10^4^  (0) | 4.53 x 10^5^  (14) | 1.94 x 10^5^  (6) | 3.236 x 10^4^ |
| Cassiar AX (1981) | 5.30 x 10^5^  (102) | 4.99 x 10^5^  (96) | 4.99 x 10^5^  (96) | 8.31 x 10^4^  (16) | 5.20 x 10^3^  (1) | 3.64 x 10^4^  (7) | 5.20 x 10^3^  (1) | 1.61 x 10^5^  (31) | 6.24 x 10^4^  (12) | 5.196 x 10^3^ |
| Cassiar AY (1984) | 6.01 x 10^6^  (188) | 5.63 x 10^6^  (176) | 5.59 x 10^6^  (175) | 2.88 x 10^5^  (9) | <3.20 x 10^4^  (0) | 2.56 x 10^5^  (8) | 3.20 x 10^4^  (1) | 1.09 x 10^6^  (34) | 2.24 x 10^5^  (7) | 3.196 x 10^4^ |
| Clinton Creek | 4.25 x 10^6^  (101) | 3.28 x 10^6^  (78) | 3.49 x 10^6^  (83) | 1.47 x 10^6^  (35) | 3.37 x 10^5^  (8) | 6.73 x 10^5^  (16) | 1.68 x 10^5^  (4) | 1.22 x 10^6^  (29) | 7.99 x 10^5^  (19) | 4.207 x 10^4^ |

Table S3. U.S.A., Brazilian and Zimbabwe Chrysotile: Fiber concentrations for various exposure indices for tremolite/actinolite fibers. Values are in fibers per gram of chrysotile. Numbers in parentheses are the numbers of fibers on which the numerical concentrations are based.

|  | Fibers  >5 µm | NIOSH | ISO | Chatfield Extra- Criteria | Stanton | Berman &  Crump | Lippmann Mesothelioma | Lippmann  Lung Cancer | Lippmann Lung Cancer with Chatfield Restriction | Analytical Sensitivity |
| --- | --- | --- | --- | --- | --- | --- | --- | --- | --- | --- |
| Chrysotile Source | | | | | | | | | | |
| Vermont 60 | 1.07 x 10^7^  (100) | 1.03 x 10^7^  (96) | 9.96 x 10^6^  (93) | 5.35 x 10^5^  (5) | 2.14 x 10^5^  (2) | 2.14 x 10^5^  (2) | <1.08 x 10^5^  (0) | 2.57 x 10^6^  (24) | 2.14 x 10^5^  (2) | 1.071 x 10^5^ |
| Vermont H1 | 1.71 x 10^6^  (41) | 1.46 x 10^6^  (35) | 1.46 x 10^6^  (35) | 2.08 x 10^5^  (5) | 8.32 x 10^4^  (2) | 1.66 x 10^5^  (4) | <4.17 x 10^4^  (0) | 4.16 x 10^5^  (10) | 1.66 x 10^5^  (4) | 4.162 x 10^4^ |
| Minaçu, Brazil  CB-4T | 2.04 x 10^4^  (1) | 2.04 x 10^4^  (1) | 2.04 x 10^4^  (1) | <2.05 x 10^4^  (0) | <2.05 x 10^4^  (0) | <2.05 x 10^4^  (0) | <2.05 x 10^4^  (0) | <2.05 x 10^4^  (0) | <2.05 x 10^4^  (0) | 2.040 x 10^4^ |
| Minaçu, Brazil  CB-7TF | 1.90 x 10^5^  (10) | 1.90 x 10^5^  (10) | 1.71 x 10^5^  (9) | <1.91 x 10^4^  (0) | <1.91 x 10^4^  (0) | 1.90 x 10^4^  (1) | <1.91 x 10^4^  (0) | 9.52 x 10^4^  (5) | <1.91 x 10^4^  (0) | 1.904 x 10^4^ |
| Zimbabwe C&G1619 | 5.70 x 10^4^  (11) | 5.70 x 10^4^  (11) | 5.70 x 10^4^  (11) | <5.19 x 10^3^  (0) | <5.19 x 10^3^  (0) | <5.19 x 10^3^  (0) | <5.19 x 10^3^  (0) | 1.04 x 10^4^  (2) | <5.19 x 10^3^  (0) | 5.183 x 10^3^ |
| Zimbabwe C&G#1 | 1.70 x 10^5^  (10) | 1.53 x 10^5^  (9) | 1.53 x 10^5^  (9) | <1.70 x 10^4^  (0) | <1.70 x 10^4^  (0) | <1.70 x 10^4^  (0) | <1.70 x 10^4^  (0) | 3.39 x 10^4^  (2) | <1.70 x 10^4^  (0) | 1.695 x 10^4^ |

Table S4. Coalinga Chrysotile: Fiber concentrations for various exposure indices for tremolite/actinolite fibers. Values are in fibers per gram of chrysotile. Numbers in parentheses are the numbers of fibers on which the numerical concentrations are based.

|  | Fibers  >5 µm | NIOSH | ISO | Chatfield Extra- Criteria | Stanton | Berman  &  Crump | Lippmann Mesothelioma | Lippmann  Lung Cancer | Lippmann Lung Cancer with Chatfield Restriction | Analytical Sensitivity |
| --- | --- | --- | --- | --- | --- | --- | --- | --- | --- | --- |
| Chrysotile Source | | | | | | | | | | |
| Coalinga  Mean of 5 Samples | 3.90 x 10^3^  (3) | 3.90 x 10^3^  (3) | 3.90 x 10^3^  (3) | <1.31 x 10^3^  (0) | <1.31 x 10^3^  (0) | <1.31 x 10^3^  (0) | <1.31 x 10^3^  (0) | <1.31 x 10^3^  (0) | <1.31 x 10^3^  (0) | 1.301 x 10^3^ |

Table S5. UICC Chrysotile: Fiber concentrations for various exposure indices for tremolite/actinolite fibers and Amosite fibers. Values are in fibers per gram of chrysotile. Numbers in parentheses are the numbers of fibers on which the numerical concentrations are based.

|  | Fibers  >5 µm | NIOSH | ISO | Chatfield Extra- Criteria | Stanton | Berman &  Crump | Lippmann Mesothelioma | Lippmann  Lung Cancer | Lippmann Lung Cancer with Chatfield Restriction | Analytical Sensitivity |
| --- | --- | --- | --- | --- | --- | --- | --- | --- | --- | --- |
| Chrysotile Sample | | | | | | | | | | |
| UICC-A  Tremolite/Actinolite (Mean of 3 Samples) | 1.53 x 10^5^  (15) | 1.53 x 10^5^  (15) | 1.53 x 10^5^  (15) | <1.03 x 10^4^  (0) | <1.03 x 10^4^  (0) | <1.03 x 10^4^  (0) | <1.03 x 10^4^  (0) | 1.02 x 10^4^  (1) | <1.03 x 10^4^  (0) | 1.023 x 10^4^ |
| UICC-A  Amosite  (Mean of 3 Samples) | 1.27 x 10^6^  (124) | 9.31 x 10^5^  (91) | 9.31 x 10^5^  (91) | 4.50 x 10^5^  (44) | 1.23 x 10^5^  (12) | 2.25 x 10^5^  (22) | 1.02 x 10^5^  (10) | 3.38 x 10^5^  (33) | 2.25 x 10^5^  (22) |  |
| UICC-B  Tremolite/Actinolite (Mean of 3 Samples) | 1.17 x 10^7^  (504) | 8.98 x 10^6^  (386) | 8.87 x 10^6^  (381) | 2.51 x 10^6^  (108) | 1.00 x 10^6^  (43) | 1.16 x 10^6^  (50) | 1.23 x 10^6^  (53) | 1.84 x 10^6^  (79) | 7.68 x 10^5^  (33) | 2.328 x 10^4^ |
| UICC-B  Amosite  (Mean of 3 Samples) | 5.12 x 10^5^  (22) | 3.72 x 10^5^  (16) | 3.72 x 10^5^  (16) | 2.09 x 10^5^  (9) | 1.16 x 10^5^  (5) | 9.31 x 10^4^  (4) | 2.33 x 10^4^  (1) | 2.33 x 10^5^  (10) | 1.40 x 10^5^  (6) |  |

Table S6. Balangero Chrysotile: Fiber concentrations for various exposure indices for diopside fibers and tremolite/actinolite fibers. Values are in fibers per gram of chrysotile. Numbers in parentheses are the numbers of fibers on which the numerical concentrations are based.

|  | Fibers  >5 µm | NIOSH | ISO | Chatfield Extra- Criteria | Stanton | Berman &  Crump | Lippmann Mesothelioma | Lippmann  Lung Cancer | Lippmann Lung Cancer with Chatfield Restriction | Analytical Sensitivity |
| --- | --- | --- | --- | --- | --- | --- | --- | --- | --- | --- |
| Chrysotile Source | | | | | | | | | | |
| Balangero  Diopside  (Mean of 3 Samples) | 2.34 x 10^7^  (404) | 2.10 x 10^7^  (363) | 2.07 x 10^7^  (358) | 6.31 x 10^6^  (109) | 1.45 x 10^6^  (25) | 3.41 x 10^6^  (59) | 5.79 x 10^5^  (10) | 1.02 x 10^7^  (177) | 4.86 x 10^6^  (84) | 5.787 x 10^4^ |
| Balangero  Tremolite/Actinolite  (Mean of 3 Samples) | 4.05 x 10^5^  (7) | 2.31 x 10^5^  (4) | 2.31 x 10^5^  (4) | 1.16 x 10^5^  (2) | 5.79 x 10^4^  (1) | 5.79 x 10^4^  (1) | 5.79 x 10^4^  (1) | 1.16 x 10^5^  (2) | <5.79 x 10^4^  (0) |  |
